# A New Pyrimidine Schiff Base with Selective Activities against *Enterococcus faecalis* and Gastric Adenocarcinoma

**DOI:** 10.3390/molecules26082296

**Published:** 2021-04-15

**Authors:** Marcin Stolarczyk, Aleksandra Wolska, Aleksandra Mikołajczyk, Iwona Bryndal, Jerzy Cieplik, Tadeusz Lis, Agnieszka Matera-Witkiewicz

**Affiliations:** 1 Department of Organic Chemistry, Faculty of Pharmacy, Wrocław Medical University, 211A Borowska, 50-556 Wroclaw, Poland; marcin.stolarczyk@umed.wroc.pl; 2 Screening Laboratory of Biological Activity Tests and Collection of Biological Material, Faculty of Pharmacy, Wroclaw Medical University, 211A Borowska, 50-556 Wroclaw, Poland; aleksandra.wolska@umed.wroc.pl (A.W.); aleksandra.mikolajczyk@umed.wroc.pl (A.M.); 3 Department of Drugs Technology, Faculty of Pharmacy, Wroclaw Medical University, 211A Borowska, 50-556 Wroclaw, Poland; iwona.bryndal@umed.wroc.pl (I.B.); jerzy.cieplik@umed.wroc.pl (J.C.); 4 Faculty of Chemistry, University of Wroclaw, Joliot-Curie Street 14, 50-383 Wroclaw, Poland; tadeusz.lis@chem.uni.wroc.pl

**Keywords:** antibacterial activity, anticancer activity, apoptosis, pyrimidines, Schiff bases

## Abstract

*Enterococcus faecalis* is known as a significant nosocomial pathogen due to its natural resistance to many antibacterial drugs. Moreover, it was found that *E. faecalis* infection causes inflammation, production of reactive oxygen species, and DNA damage to human gastric cancer cells, which can induce cancer. In this study, we synthesized and tested the biological activity of a new Schiff base, 5-[(4-ethoxyphenyl)imino]methyl-*N*-(4-fluorophenyl)-6-methyl-2-phenylpyrimidin-4-amine (**3**), and compared its properties with an analogous amine (**2**). In the biological investigation, **3** was found to have antibacterial activity against *E. faecalis* 29212 and far better anticancer properties, especially against gastric adenocarcinoma (human Caucasian gastric adenocarcinoma), than **2**. In addition, both derivatives were non-toxic to normal cells. It is worth mentioning that **3** could potentially inhibit cancer cell growth by inducing cell apoptosis. The results suggest that the presence of the –C=N– bond in the molecule of **3** increases its activity, indicating that 5-iminomethylpyrimidine could be a potent core for further drug discovery research.

## 1. Introduction

*Enterococcus* is a large genus of gram-positive cocci traditionally considered part of the lactic acid bacteria [1]. They are ubiquitous and can be found as commensal organisms in the gastrointestinal tract of humans and animals, and also in soil, water, and plants [2]. Furthermore, their resistance to physical and chemical conditions and ability to exploit various substrates as a growth medium results in their being present also in food [3]. Generally, they are not desirable in processed meat because they prompt decaying processes. However, on the other hand, they have an important application in the fermentation products industry because of their significant role in developing peculiar organoleptic characteristics of such food products. Thus, *Enterococci* are included as part of starting cultures in cheese production [4]. It was reported that *Enterococci* were used as probiotics, but such application became a controversial issue owing to the emergence of antibiotic-resistant strains and their association with human diseases (see below) [3]. Over the years, *Enterococci* were believed to be harmless to humans and thus medically unimportant. They were recognized as a significant nosocomial pathogen due to their natural resistance to antibacterial drugs, and their ability to acquire virulence and multidrug resistance even to last-resort medicines such as linezolid, quinupristin-dalfopristin, or daptomycin [5]. *Enterococci* can cause different infections (e.g., endocarditis, bacteremia, urinary tract infections, meningitis, and wound infections). Currently, they are the leading cause of hospital-acquired infections [6]. It was reported that enterococcal cell extracts and whole organisms are capable of generating superoxide (O^2−^) [7], which can lead to oxidants like hydrogen peroxide or hydroxyl radicals that damage DNA [8] and induce genetic instability in cells [9,10]. The bacterium also activates macrophages to produce 4-hydroxy-2-nonenal, which in turn promotes colon carcinogenesis in Il10−/− mice [11]. One study demonstrated that the bacterium is significantly more often detected in stool specimens of colorectal cancer cases than in controls [12]. The nexus of the presence of *E. faecalis* and stomach carcinogenesis is critical. *Enterococci* are predominant bacteria found in the stomachs of achlorhydric mice [13]. Their presence may increase stomach cancer risk in achlorhydric individuals since the overgrowth of bacteria other than *Helicobacter pylori* in the gastric lumen is common among patients with gastric cancer [14]. It was also confirmed that *E. faecalis* infection causes inflammation, production of reactive oxygen species, and DNA damage to human gastric cancer cells. There are many similarities between infections by *E. faecalis* and *H. pylori* that can induce cancer-promoting events in humans [15,16].

**Figure 1:**
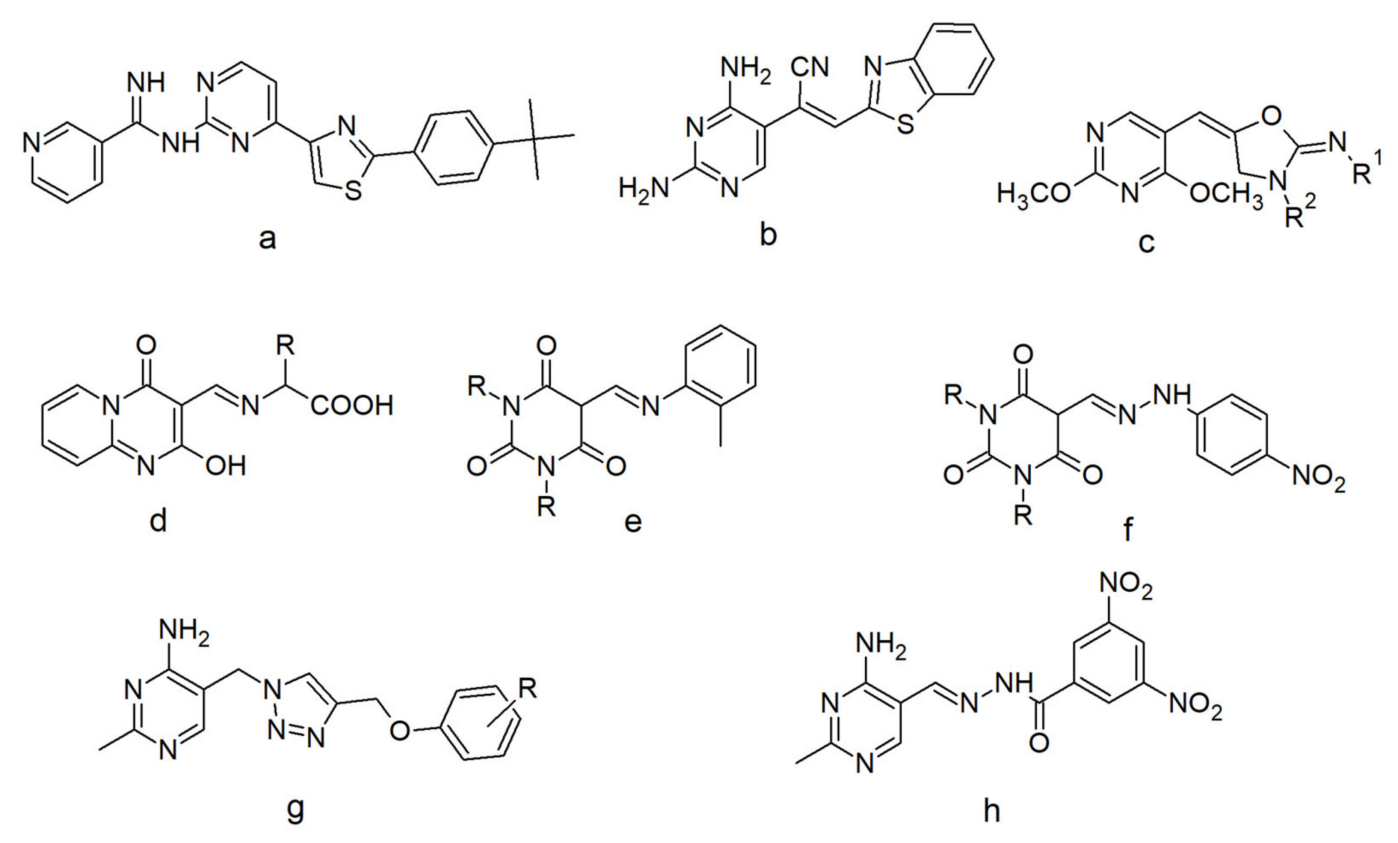
New potent antibacterial pyrimidines: derivative of 2,4-disubstitued pyrimidine (**a**); pyrimidines with the double bond of the chain at position 5 of the ring (**b**,**c**); pyrimidines containing the azomethine group at position 5 of the ring (**d**–**f**); lead structure (**g**) and the most active derivative obtained by He et al. (**h**)The azomethine group is present in such classes of compounds as imines, oximes, or hydrazones that are formed in the reaction of carbonyl compounds with amines, hydroxylamine, or hydrazine, respectively. In the chemistry of pyrimidines, the formation of this group mostly employs the reaction of aminopyrimidines with aldehydes [26]. Sometimes pyrimidine-5-carbaldehyde is also used [27]. Imines substituted at nitrogen with alkyl or aryl group are known as Schiff bases and were first described in 1864 [28]. To date, many methods for their synthesis have been described. The most common employ the removal of water, using a catalytic amount of Lewis acids and irradiation techniques [29,30,31,32]. They still arouse the interest of scientists because of the wide range of pharmacological activities they exhibit, including, among others: antimicrobial [33], anticancer [34,35], anti-inflammatory [36], and analgesic activity [37].

Pyrimidine is commonly found in nature as a constituent of nucleic acids and many other natural and synthetic compounds, including drugs. It is known as a potent pharmacophore. Many pyrimidine derivatives have been established as potentially valuable sources of antibacterial, anti-inflammatory, antitumor, and anticancer agents [17,18]. Recently, several new 2,4-disubstitued pyrimidine compounds acting against *E. faecalis* have been obtained and studied [19]. The most potent compound has a nitrogen side chain attached to the pyrimidine ring at position 2 (Figure 1a). Furthermore, strong antibacterial activity is exhibited by pyrimidines containing the double bond of the chain at position 5 of the ring. Al Neyadi et al. demonstrated that the double bond in (*E*)-2-(benzo[d]thiazol-2′-yl)-3-(2″,4″-diaminopyrimidin-5″-yl)acrylonitrile (Figure 1b) has the potential to irreversibly bind to the penicillin-binding protein (PBP) and covalently bind to the β-lactamase enzyme, which explains its capability to overcome bacterial resistance by demonstrating significant synergism when combined with amoxicillin [20]. Good bactericidal activity and low toxicity to human cells were also shown by pyrimidine-oxazolidone analogs (Figure 1c), wherein the rings are connected through a methine bridge. A molecular docking study suggested that these compounds could inhibit protein biosynthesis by binding to sites on the bacterial ribosomes [21]. Antibacterial and antifungal effects of imines of pyridi[1,2-a]pyrimidine (Figure 1d) and various amino acids were reported. Researchers found that aromatic amino acid compounds are more potent than aliphatic amino acid compounds [22]. Strong antifungal activity was also exhibited by Shiff bases (Figure 1e) and phenylhydrasones (Figure 1f) of pyrimidinetriones. Their minimum inhibitory concentration (MIC) against *C. albicans* and *C. glabrata* ranged from 1 to 8 ug/mL, and a mechanism of action connected with hindering fungal energy production was proposed [23]. By modifying the lead structure of 2-methylpyrimidine-4-ylamine derivatives containing a 1,2,3-triazole ring, He et al. substituted the benzene ring and the aminomethyl bridge at position 5 of the pyrimidine ring (Figure 1g) and obtained new, highly potent pyruvate dehydrogenase multienzyme complex E1 (PDHc E1) inhibitors with bactericidal activity. It was demonstrated that the inhibitory potency of compounds against *E. coli* PDHc E1 could be greatly enhanced when the linkage (between pyrimidine and benzene ring moiety) of the lead compound was replaced with *N*-acylhydrazone moiety. The most potent compound (Figure 1h) containing the azomethine group (–CH=N–) could selectively inhibit the enzyme activity of microorganisms with little effect on mammalian cells, and thus could be used as a novel lead structure for further optimization [24,25].

By now, our research group has synthesized a number of 6-methyl-2-phenylpyrimidin-4-amine derivatives and evaluated their antimicrobial properties. It was found that the pyrimidine amines that possess a halo group attached to the phenyl ring at the amino group at position 4 and an alkoxy group at the phenyl ring at position 5 of the 5-(aminomethyl)-6-methyl-2-phenylpyrimidin-4-aminesystem and vice versa exhibit strong antibacterial properties [38,39]. It should be mentioned that a similar impact of the substituents was also observed in the case of 1,2,3-triazolepyrimidine-based hybrids that can act as potent reversal agents against ABCB1-mediated multidrug resistance [40]. Additionally, in the study on 4-aminopyrimidine, we were mainly focused on its 5-aminomethyl derivatives and their cyclization products with aldehydes because of their interesting antimicrobial activity [41,42].

During our further research, in the reaction of the product of chlorination of [4-(4-fluoroanilino)-6-methyl-2-phenylpyrimidin-5-yl]methanol (**1**) with *p*-phenetidine, we observed on a TLC plate not only the expected 5-[(4-ethoxyanilino)methyl]-*N*-(4-fluorophenyl)-6-methyl-2-phenylpyrimidin-4-amine (**2**), but also an additional light yellow spot that turned out to be a new pyrimidine Schiff base, namely 5-[(4-ethoxyphenyl)imino]methyl-*N*-(4-fluorophenyl)-6-methyl-2-phenylpyrimidin-4-amine (**3**).

The therapeutic importance of suitably functionalized pyrimidines encouraged us to develop an innovative and effective synthesis of Schiff bases in which halo and alkoxy substituents could be arranged in a pharmacophoric pattern to display diverse pharmacological activities. Herein, we evaluated the synthesis and biological properties of a new pyrimidine Schiff base that combines the antibacterial activity against *E. faecalis* and the anticancer potential against AGS (gastric adenocarcinoma) cell lines, compared its properties with an analogous amine, and examined the structure-activity relationships.

## 2. Results

### 2.1. Chemistry

**Scheme 1:**
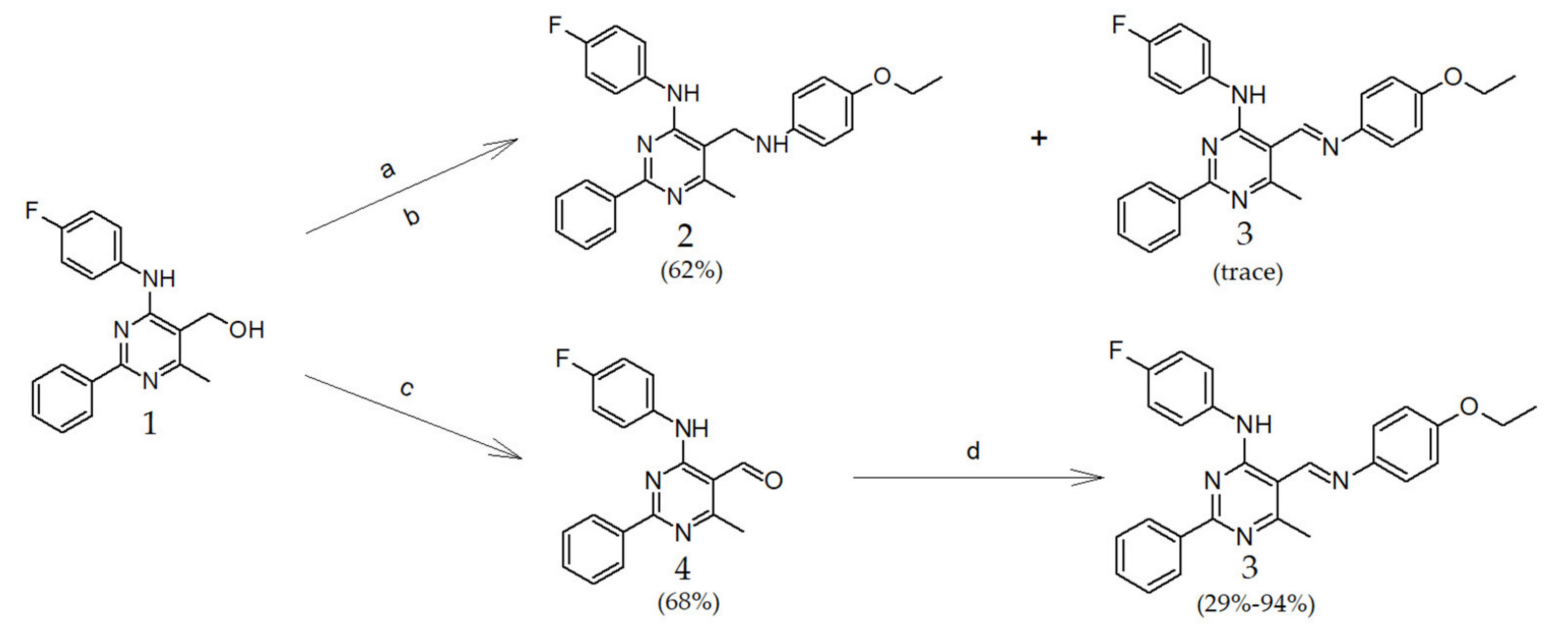
Synthesis of compounds **2–4**. Conditions: (**a**) SOCl_2_, benzene, 24 h, r.t.; (**b**) *p*-phenetidine, THF, 24 h, r.t.; (**c**) PCC, CH_2_Cl_2_, 3 h, r.t.; (**d**) *p*-phenetidine, THF, 3 h, b.p.; or *p*-phenetidine, In(OTf)_3_, THF, 24 h, r.t.; or *p*-phenetidine, In(OTf)_3_, MeOH, 24 h, b.p.

The studied compounds were synthesized as outlined in Scheme 1. The synthesis started with **1**, which was obtained and then halogenated with SOCl_2_ according to the procedure described in the literature [43]. Then the gray halogen-derivative was directly coupled with *p*-phenetidine in tetrahydrofuran (THF) at r.t. After 24 h on a TLC plate beyond the amine spot, we also observed a light yellow speck of an unidentified compound. During the separation of products via column chromatography, the additional product went with the solvent front and was collected.

The analysis of MS spectra (see Figures S1–S3 in the Supplementary Materials of this paper), which compares the isotopic distribution of the ion with the pattern (Figure S4), indicated that the unknown compound has the same structure as the proposed Schiff base **3**. The IR spectrum (Figure S5) also showed the presence of the –CH=N– group. Unfortunately, the compound amount was too small to perform other tests.

The NMR spectrum of the main product of the reaction (compound **2**, Figure S6) showed characteristic signals from the ethyl group of *p*-phenetidine (1.42, t, 3H; 4.01, q, 2H) and four additional aromatic protons. The newly formed amino group could be observed as a broad signal at about 3.60 ppm. The IR spectrum (Figure S7) showed the secondary amino group at 3295 cm^−1^. In addition, the MS spectrum (Figure S8) and elemental analysis confirmed the structure of the obtained derivative.

We found it noteworthy to examine the impact of the single versus double bond in the structures of **2** and **3** on their features and activity. Therefore, we decided to work out a method of synthesizing **3** that would enable us to obtain a sufficient amount of the compound for further investigation. The oxidation of the hydroxyl group of 1, and then coupling it with *p*-phenetidine, appeared most practical. Pyridinium chlorochromate (PCC) was selected as the oxidizing agent due to its stability, selectivity, and mild reaction conditions [44]. The reaction was carried out in CH_2_Cl_2_ at room temperature for 3 h to obtain aldehyde **4**. The NMR spectrum (Figure S9) showed the proton of the aldehyde group at 10.43 ppm. On the IR spectrum (Figure S10), the carbonyl group could be seen at 1630 cm^−1^. The MS spectrum (Figure S11) and elemental analysis were also in line with expectations. The aldehyde **4** was coupled with *p*-phenetidine in three different ways to obtain Schiff base **3**. The reaction in THF in the presence of a catalytic amount of indium(III) trifluoromethanesulfonate (In(OTf)_3_) as Lewis acid at r.t. turned out to be the most effective. The NMR spectrum (Figure S12) showed characteristic signals from the ethyl group of *p*-phenetidine (1.46, t, 3H; 4.09, q, 2H) and additional aromatic protons. Other analyses also confirmed the structure of compound **3**. The IR spectra of the compound obtained in the first reaction and through the coupling of aldehyde **4** with *p*-phenetidine were identical (compare Figures S5 and S13). The analysis of the MS spectrum of **3** showed a signal at 427.1882 and was in line with expectations (Figure S14).

### 2.2. X-ray Structural Studies

Single-crystal X-ray diffraction was used to determine the crystal structures of compounds **2** and **3**. Details of the data collections, analyses, and refinements of the studied crystals are given in Table S1. A comparison of selected geometrical parameters is presented in Table S2, Supplementary Materials of this paper.

**Figure 2:**
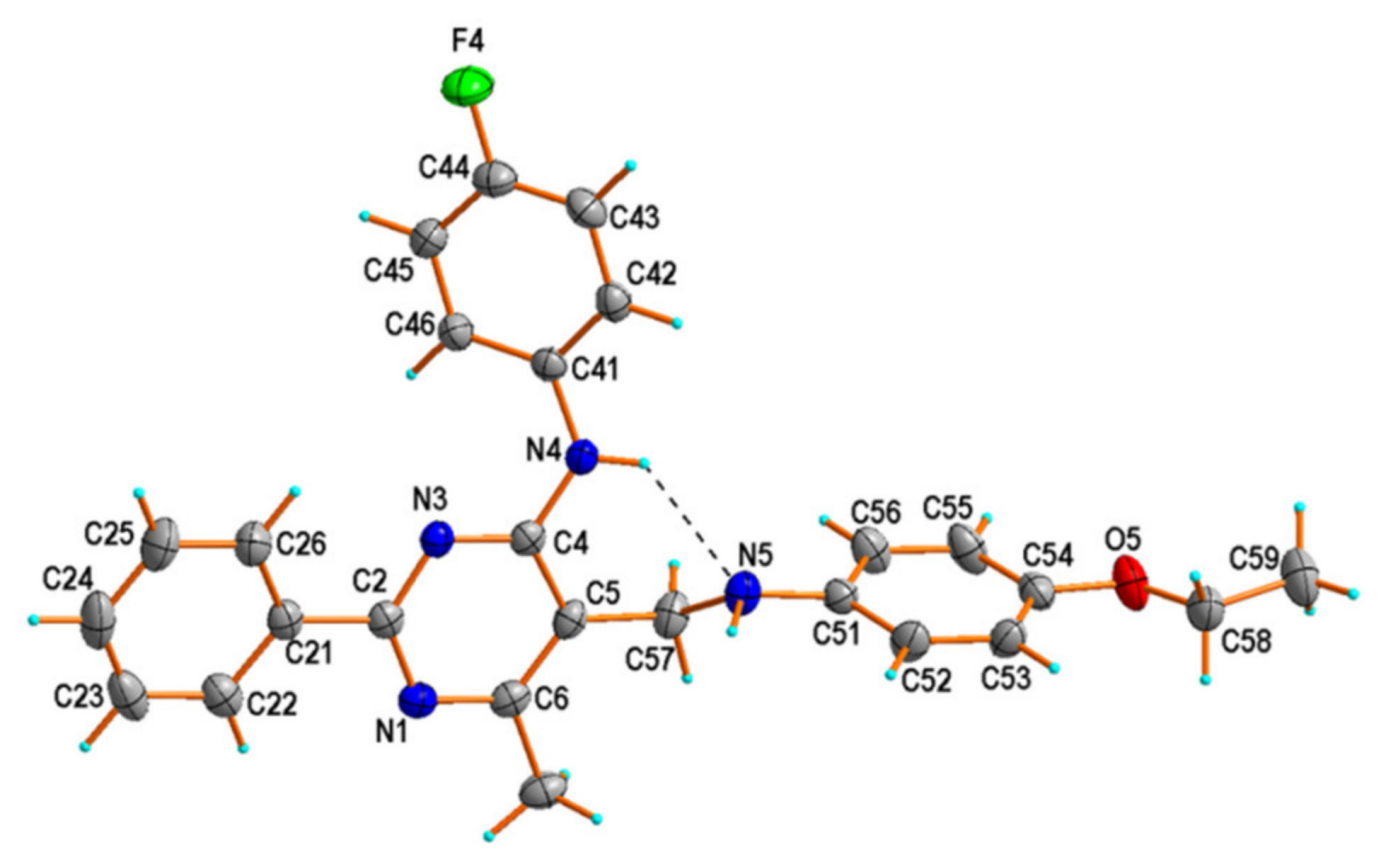
X-ray molecular structure of **2** with the atom-numbering scheme. Displacement ellipsoids are drawn at the 50% probability level. H atoms are shown as small spheres of arbitrary radii. The dashed line (in black) indicates the N–H⋯N hydrogen bond.

The crystal structure analysis revealed that compound 2 belongs to the orthorhombic crystal system with the Pca21 space group (Z = 4), and crystallizes with one molecule in the asymmetric unit (Figure 2).

There is an intramolecular N–H⋯N hydrogen bond between N4–H4 and N5 of both amine groups (Table 1), which closes a six-membered ring with an S(6) motif [45]. Such an intramolecular hydrogen bond, with a similar synthon, can be found in previously studied crystal structures of 2-phenylpyrimidine-4-amine derivatives [46,47,48]. The N4⋯N5 distance [2.9365 (18) Å] is generally longer in **2** than in other such derivatives [47,48]; however, it is comparable with 2.940 (3) Å observed in one polymorphic form (denoted as Ia) of *N*-(4-chlorophenyl)-5-[(4-chlorophenyl)aminomethyl]-6-methyl-2-phenylpyrimidin-4-amine [46]. The dihedral angles describe the conformation of the molecule between the pyrimidine ring plane and those of the phenyl ring attached to atom C2 and two other aryl rings of the (4-fluorophenyl)amino or (4-ethoxyphenyl)aminomethyl groups, attached to atoms C4 or C5 of the pyrimidine ring. The dihedral angles are 8.9 (2), 36.8 (2), and 79.2 (2)°, respectively.

**Figure 3:**
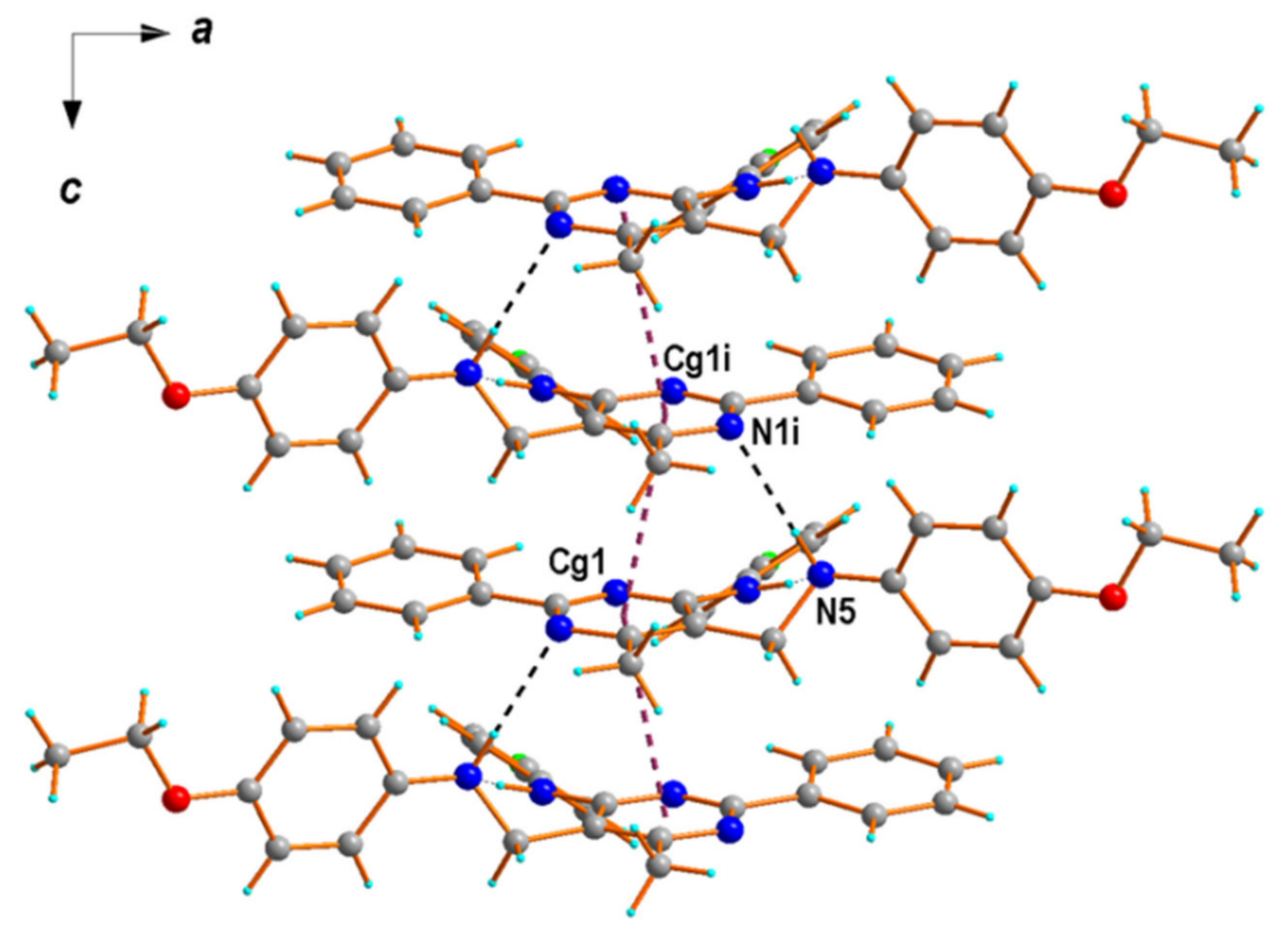
Part of the crystal structure of **2**, showing the formation of the one-dimensional chain through intermolecular interactions with N–H⋯N (in black) and π-π (in plum). Cg1 is the centroid of the N1–C6 ring. Symmetry code is the same as in Table 1.

The molecules of **2** are linked by an intermolecular N–H⋯N hydrogen bond involving N5 amine nitrogen as a donor and N1 pyrimidine nitrogen of (− x + 1/2, y, z − 1/2) as an acceptor (Table 1), which are building a molecular chain in the direction of the c-axis. Within such chains, one can identify aromatic π-π stacking interactions (with a centroid⋯centroid separation of 3.71 Å) between pyrimidine rings (Figure 3). Neighboring chains are linked to each other by C−H⋯O and C−H⋯F contacts, generating a three-dimensional (3D) network (Figure S15, Supplementary Materials of this paper).

**Figure 4:**
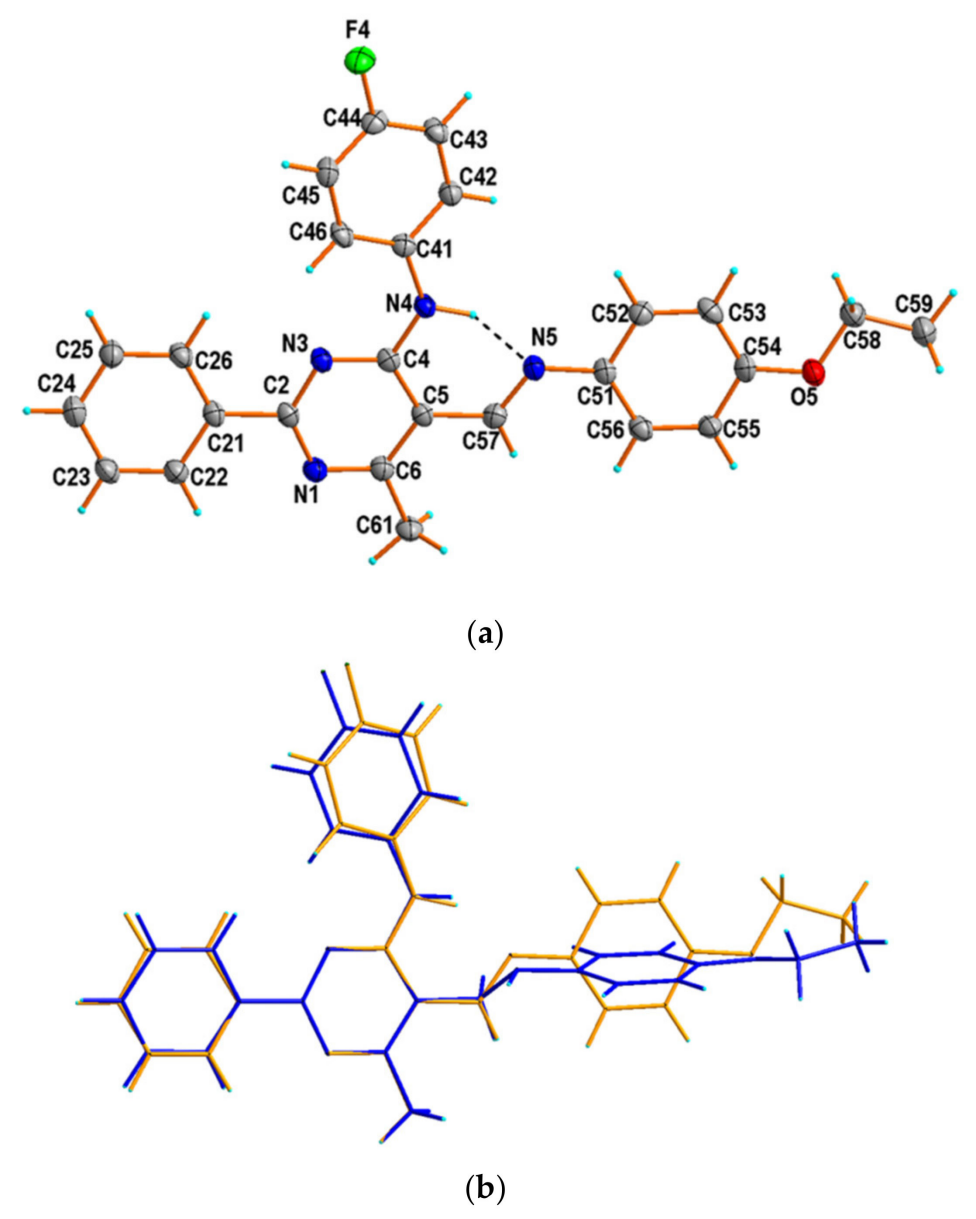
(**a**) X-ray molecular structure of **3** with the atom-numbering scheme. Displacement ellipsoids are drawn at the 50% probability level. H atoms are shown as small spheres of arbitrary radii. The dashed line (in black) indicates the intramolecular N–H⋯N hydrogen bond. (**b**) Overlapping in the structure of **2** (in blue) and **3** (in light orange); the common reference points are N1, C2, N3, C4, C5, and C6 atoms of the pyrimidine rings.

The structure determination revealed that compound **3** crystallizes in the monoclinic space group P21/c (Z = 4), with one molecule in the asymmetric unit (Figure 4a). The molecule of **3**, compared to the molecule of **2**, differs in the presence of an imine bond [1.288 (2) Å] instead of an amine bond [1.461 (2) Å] between C57 and N5 atoms, which is also reflected by a change in orientation of the benzene rings attached to the pyrimidine ring (Figure 4b). In the case of **3**, the aryl rings attached to atoms C2 and C4 of the pyrimidine ring are only slightly twisted with respect to the pyrimidine ring plane, with the dihedral angles of 10.3 (3) and 15.2 (3)°, respectively. The pyrimidine and 4-ethoxyphenyl rings in **3** adopt an E configuration along the imino functional group, and both the planes of the pyrimidine and the phenyl moieties are twisted only at 8.6 (3)° to each other, respectively. This orientation is probably stabilized by the intramolecular N–H⋯N hydrogen bond between N4–H4 and N5 of amine and imine groups (Table 1), similar to **2**. However, the N4⋯N5 distance of 2.664 (2) Å is shortened compared to 2.937 (2) Å observed in **2**.

**Figure 5:**
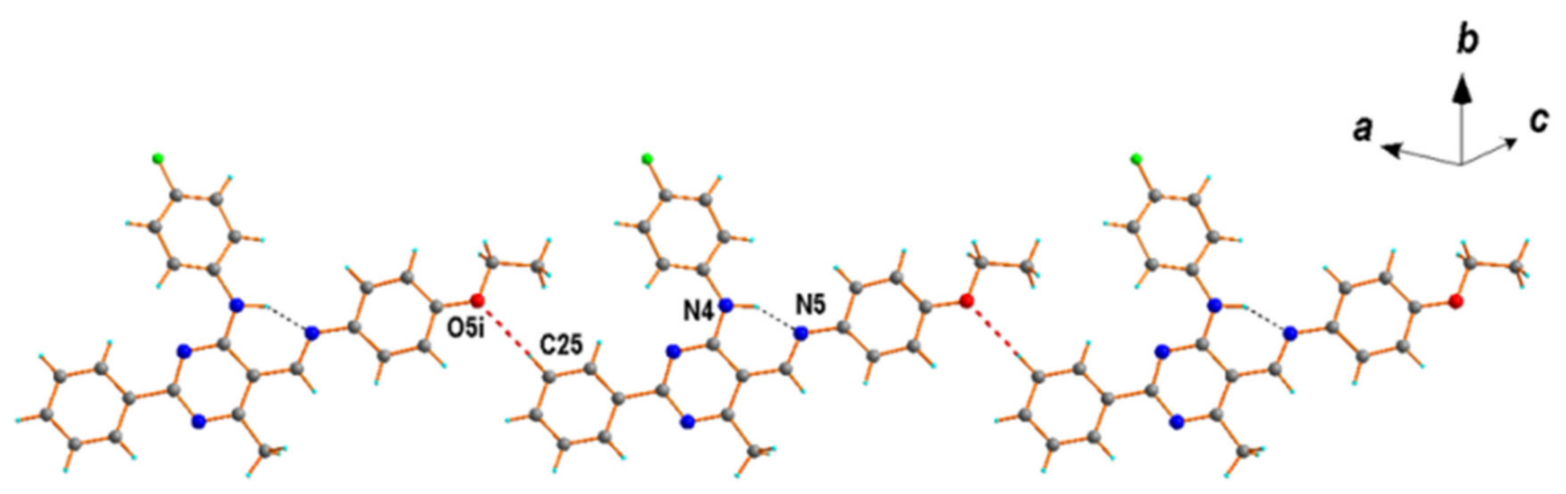
Part of the crystal structure of **3** showing the formation of the one-dimensional chain through C–H⋯O (in red) intermolecular interactions. The dashed lines (in black) indicate intramolecular N–H⋯N hydrogen bonds. Symmetry code is the same as in Table 1.

The molecules of **3** are linked by an intermolecular C–H⋯O hydrogen bond, involving C5 aromatic carbon as a donor and O5 ethoxy oxygen of (x + 1, y, z − 1) as an acceptor, and build molecular chains (Table 1, Figure 5). A 2D hydrogen-bonded network is created by the connection of neighboring chains through C−H⋯F hydrogen-bonding interactions (Figure S16).

### 2.3. Cells and Cytotoxicity Assay

#### 2.3.1. Proliferation Inhibition Caused by **2** and **3**

**Figure 6:**
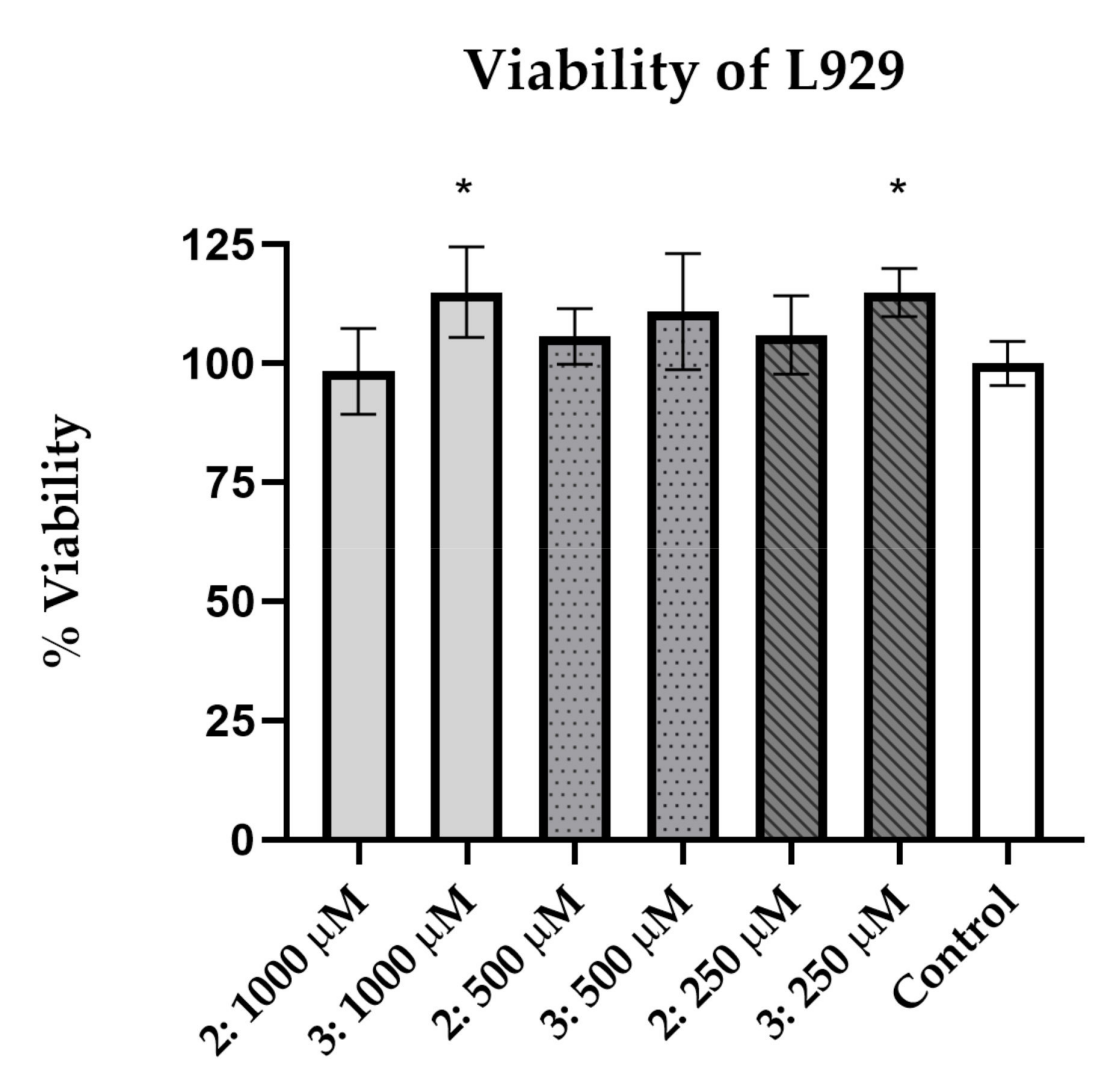
Viability of L929 cells after treatment with **2** and **3**. * Significant difference from control (*p* < 0.05).

Regarding primary screening, a neutral red uptake assay was performed using the L-929 cell line. Results are shown in Figure 6. An enhancement of cell viability for **3** was observed (*p* < 0,05; viability for 1000 µM = 115%, 500 µM = 111%, 250 µM = 115%), and **2** did not significantly affect L-929 survival. Presented results are good input data for further investigation toward potential cytotoxic features of the analyzed compounds.

**Figure 7:**
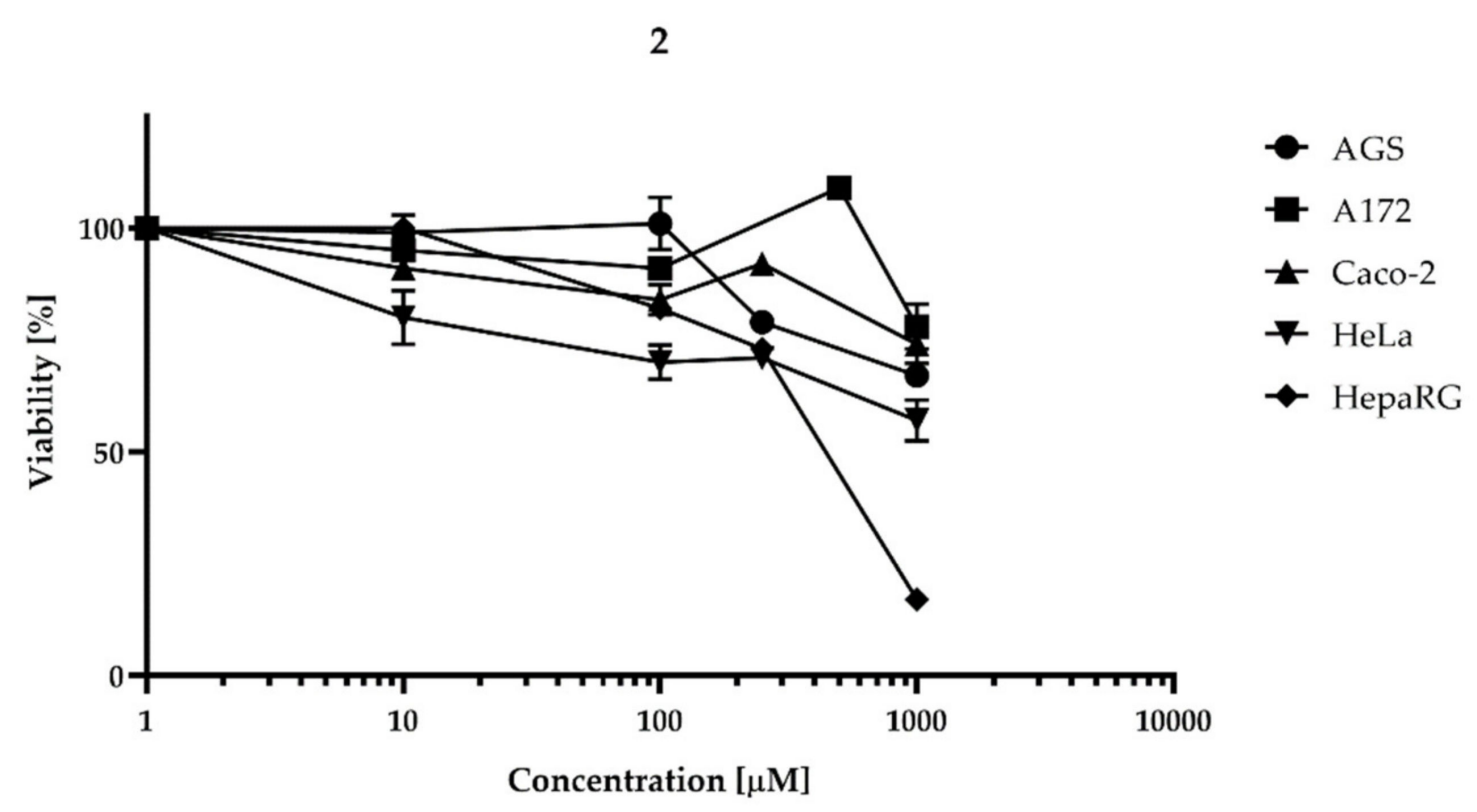
Viability changes after 72 h exposure to **2**. Concentrations applied to cells: 1000, 250, 100, and 10 µM for Caco-2, A172, HepaRG, and HeLa; concentrations for AGS cell line: 1000, 500, 100, and 10 µM. Concentrations were logarithmized, and viability was calculated as percentages.

**Figure 8:**
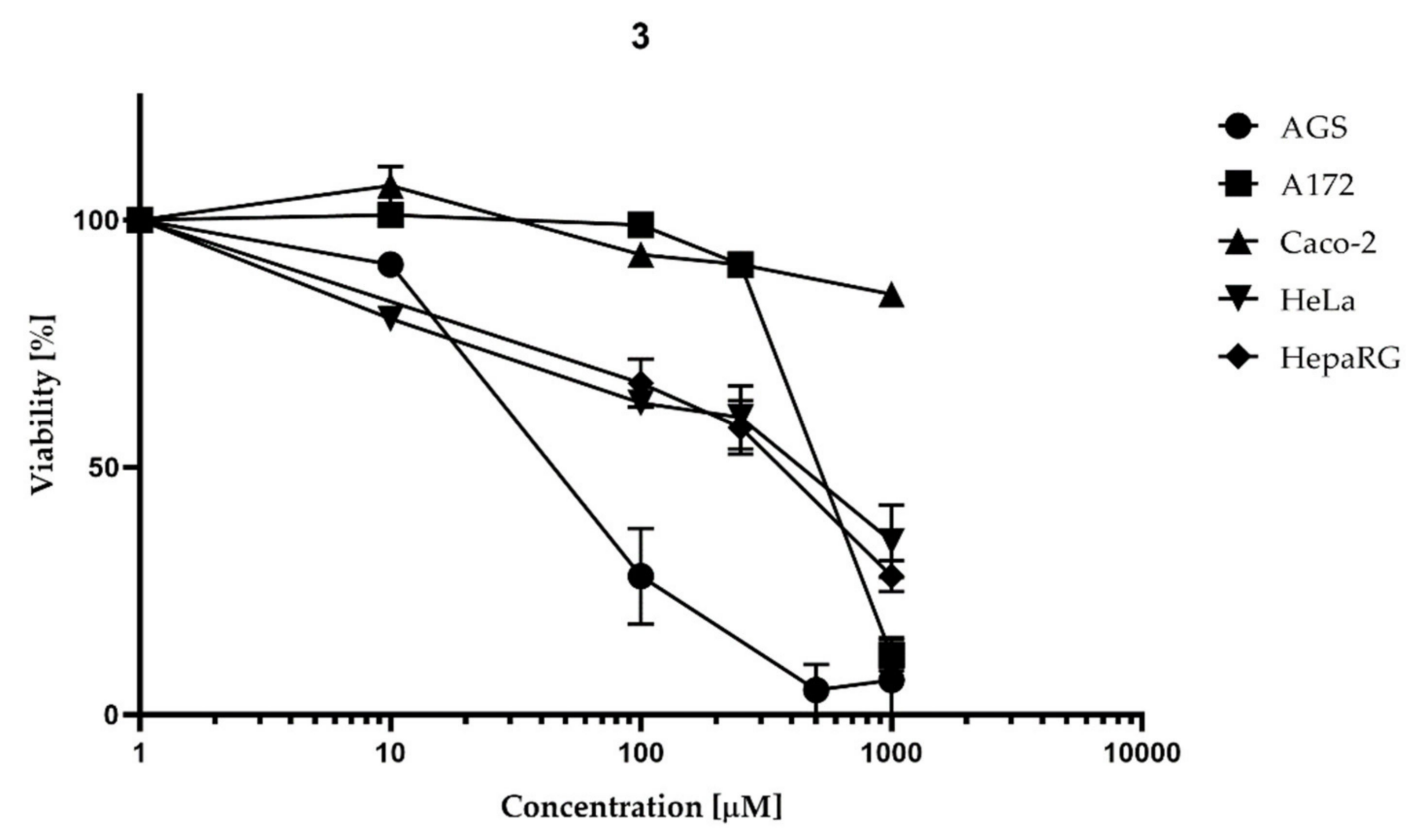
Viability changes after 72 h exposure to **3**. Concentrations applied to cells: 1000, 250, 100, and 10 µM for Caco-2, A172, HepaRG, and HeLa; concentrations for AGS cell line: 1000, 500, 100, and 10 µM. Concentrations were logarithmized, and viability was calculated as percentages.

The neutral red uptake assay was further used to investigate the cytotoxic effect for neoplastic cell lines. Obtained results for **2** show selective cytotoxicity to HepaRG (IC50 404.2 µM). For other cell lines, **2** decreased viability when applied in high concentrations (100–1000 µM), but IC50 was not reached (Figure 7), while **3** expressed sufficient activity to calculate IC50 for AGS (IC50 = 50.24 µM), HepaRG (IC50 = 312.7 µM), HeLa (IC50 = 355.4 µM), and A172 (IC50 = 526.2 µM) (Figure 8).

**Figure 9:**
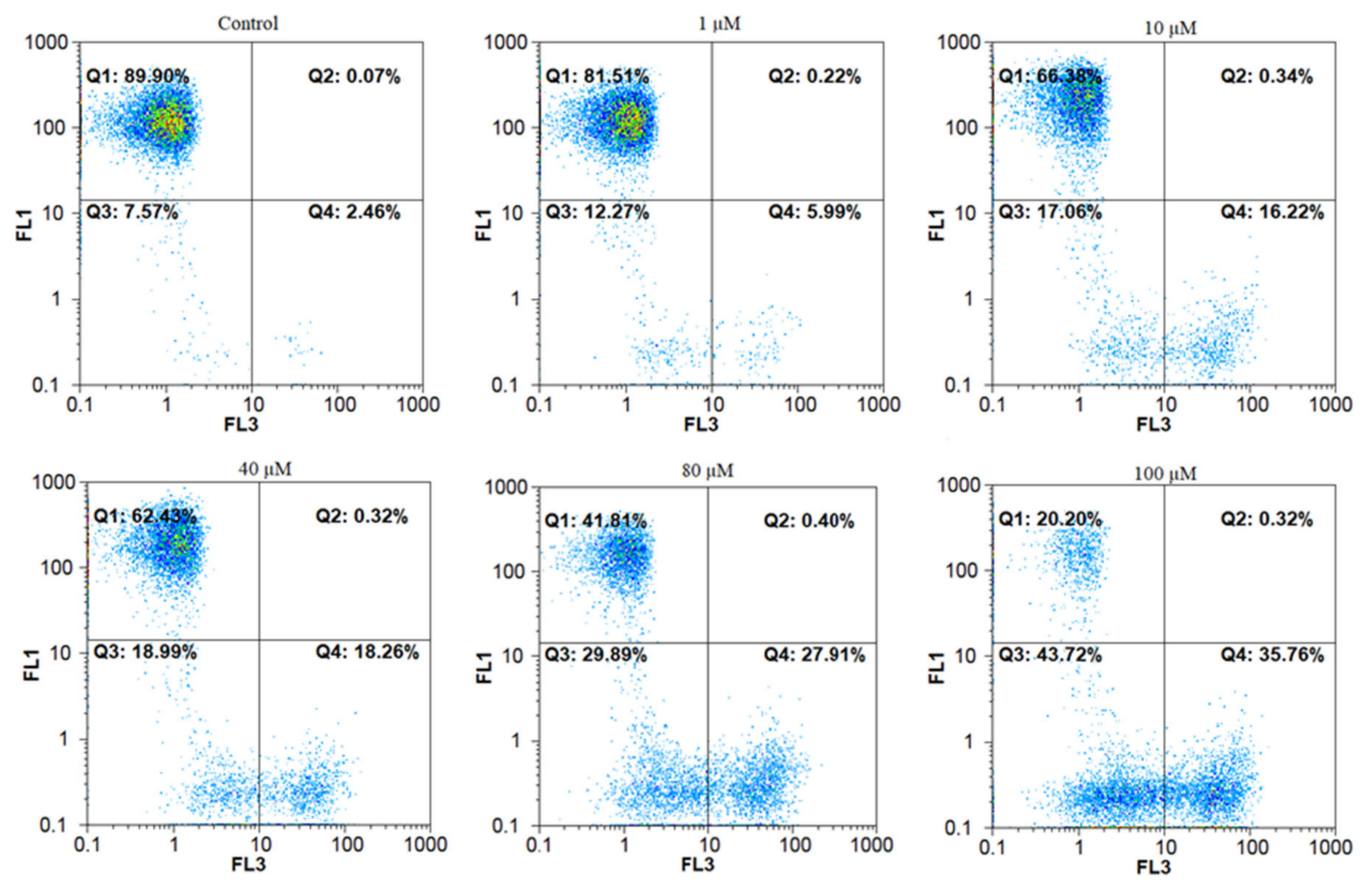
The cytotoxic effect after AGS cell line treatment with **3** tested using flow cytometry. FL1 channel represents 536/40 filter, FL3 channel 675/20. Fluorochromes were excited by blue laser (488/50 nm).

Flow cytometry was performed for the AGS cell line after incubation with **3** within the 1, 10, 40, 80, and 100 µM concentration ranges. Results are shown in Figure 9. FDA and PI were used as indicators for viable and nonviable cells. The first quadrant (Q1) contains cells stained with FDA, which is converted to fluorescein by esterases in viable cells.

Region four (Q4) consists of dead cells stained with PI due to plasma membrane integrity loss. Unstained cells are located in the third region (Q3), and double-stained cells are in the second quadrant (Q2). Quantitative analyses were performed from at least 20,000 events per sample. The number of cells in the first quadrant decreased from 89.90% to 20.20% for 0 to 100 µM, respectively. Subsequently, the number of cells in the third quadrant increased from 7.57% to 43.72%, and in the fourth region, it rose from 2.46% to 35.76% for 0 to 100 µM. The exact IC50 value calculated in the GraphPad Prism software was 53.02 µM. The results were referred to control with 1% DMSO, which was considered 100% viable.

#### 2.3.2. Morphological Changes Induced by Imine **3**

Imine **3** displays sufficient activity to calculate IC50 for AGS (IC50m = 50.24 µM), HepaRG (IC50 = 312.7 µM), HeLa (IC50 = 355.4 µM), and A172 (IC50 = 526.2 µM). Flow cytometry of the most promising AGS cell line confirmed IC50 (53.02 µM) and showed a high percentage of unstained cells (43.72% for 100 µM).

**Figure 10:**
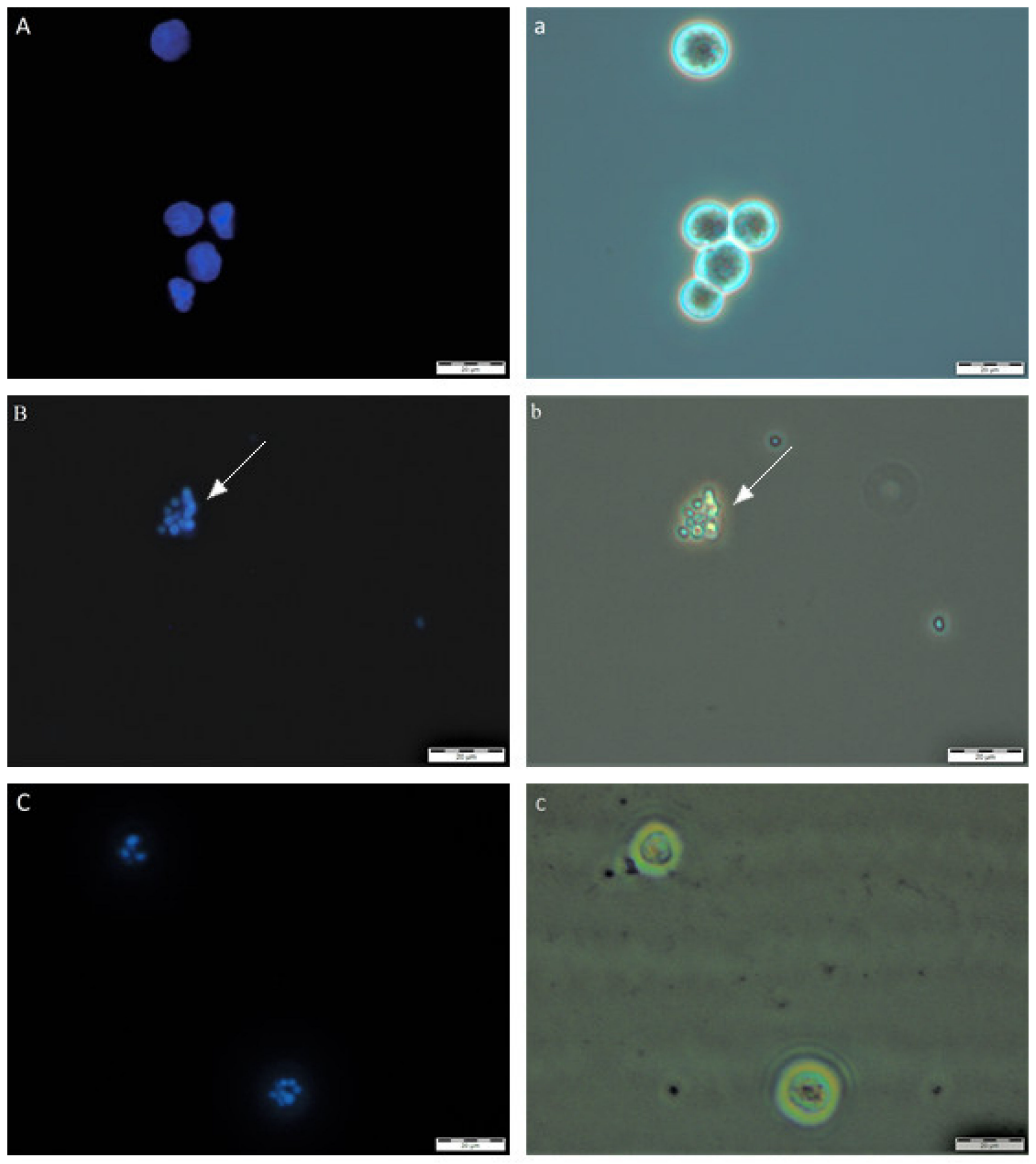
Analysis of cell and nucleus morphology of the AGS cell line treated with 50 µM of **3** after 72 h. Pictures were taken under fluorescence microscopy with 400× magnification; Hoechst 33342 dye was used in the experiment. Pictures from the UV filter are described in capital letters, pictures of the contrast phase are indicated with small letters. **A**,**a**—cell characteristics in negative control (medium with 1% DMSO); **B**,**b**—cells after treatment; **C**,**c**—morphological and nucleus changes in positive control (1 µM staurosporine).

Cellular morphology observation after treatment with **3** was performed for AGS due to the highest viability decreasing activity on this cell line in the area of potentially promising applied concentration. Examination of cells was performed in IC50 (50 µM) under a fluorescence microscope with contrast phase and UV filter using Hoechst 33342 (Figure 10). Cells after treatment are shown in Figure 10B,b. Additionally, after 72 h, cells treated with 50 µM broke apart into apoptotic bodies, suggesting that compound **3** inhibits the growth of AGS cells by inducing apoptosis. The apoptotic bodies are marked with arrows. For comparison, intact cells with a round nucleus from negative control are presented in Figure 10A,a. Shrunken cells’ bodies and fragmented DNA after positive control treatment (1 µM staurosporine) are shown in Figure 10C,c.

#### 2.3.3. Cell Death

**Figure 11:**
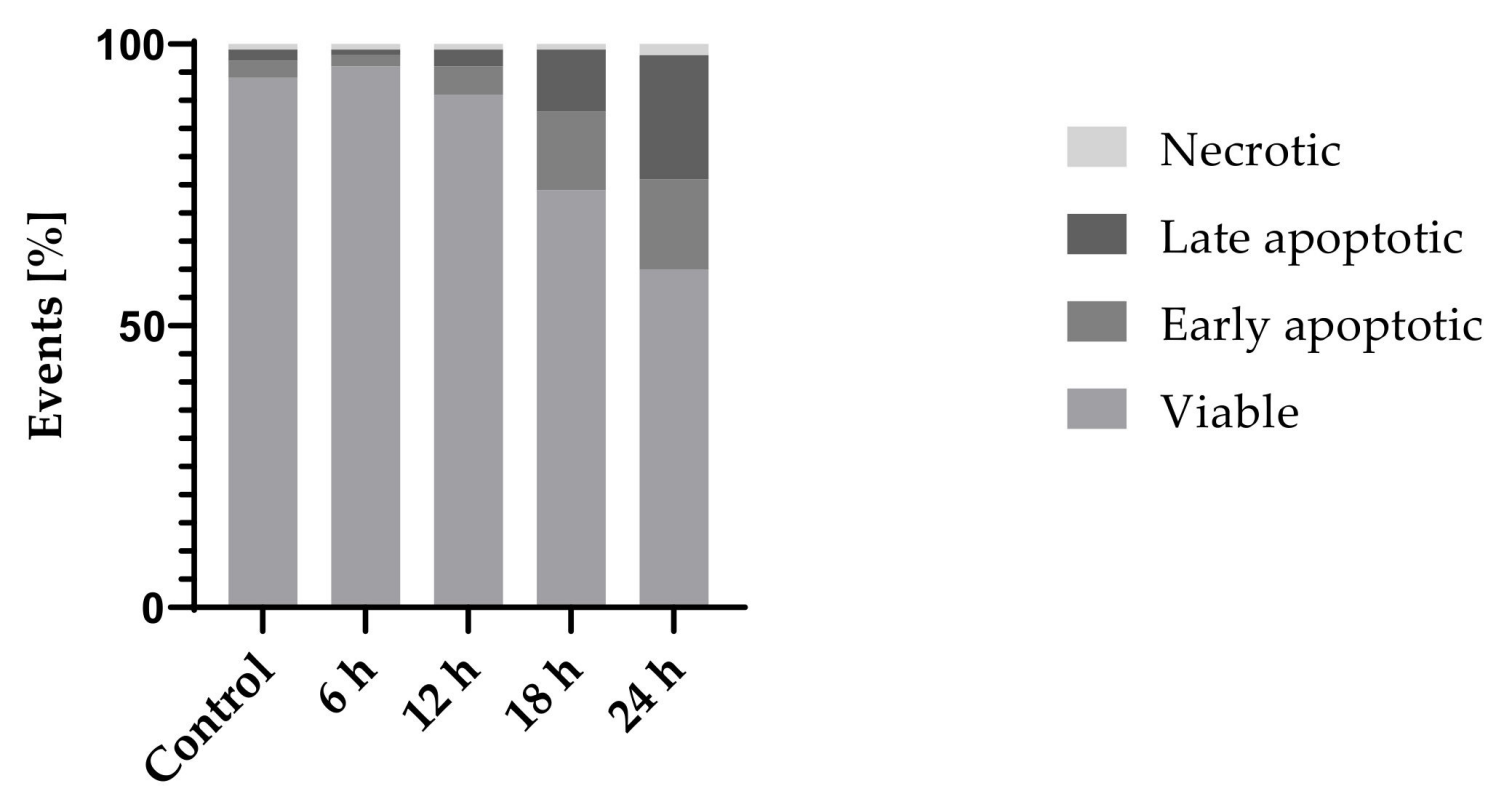
Alexa Fluor 488-annexin V and PI staining of the AGS cell line after exposure to **3** at 250 µM concentration.

To confirm the cell death pathway, annexin V/PI staining was performed. Testing with annexin V allows observing the early stage of apoptosis when the phosphatidylserine is translocated from the inner to the outer cell membrane [49]. Propidium iodide can enter cells with cell membrane integrity loss; therefore, double-stained cells can be marked as late apoptotic. The results after 24 h incubation with **3** (Figure 11) showed an increasing number of early (16.18%) and late (21.58%) apoptotic cells. Other changes occurring in the executive pathway of cell death include degradation of chromosomal DNA [50]. Unstained cells were considered viable, early apoptotic cells were stained with Alexa Fluor 488 annexin V, late apoptotic cells were double-stained, and necrotic cells were stained with PI (Figure 11). Cells incubated with solvent (1% DMSO) were used as a negative control. Positive control of apoptosis was 1 µM staurosporine. Percentage of viable cells decreased from 93.57% in the control group to 60.02% after 24 h incubation, with an increase in the early apoptotic fraction (from 3.24% in control to 16.18% after 24 h incubation) and the late apoptotic fraction (from 2.48% in control to 21.58% after 24 h incubation). The fraction of necrotic cells (stained with PI only) slightly increased (0.71% in the control group and 2.22% in the group incubated with **3** for 24 h).

#### 2.3.4. Genotoxicity/DNA Damage

**Figure 12:**
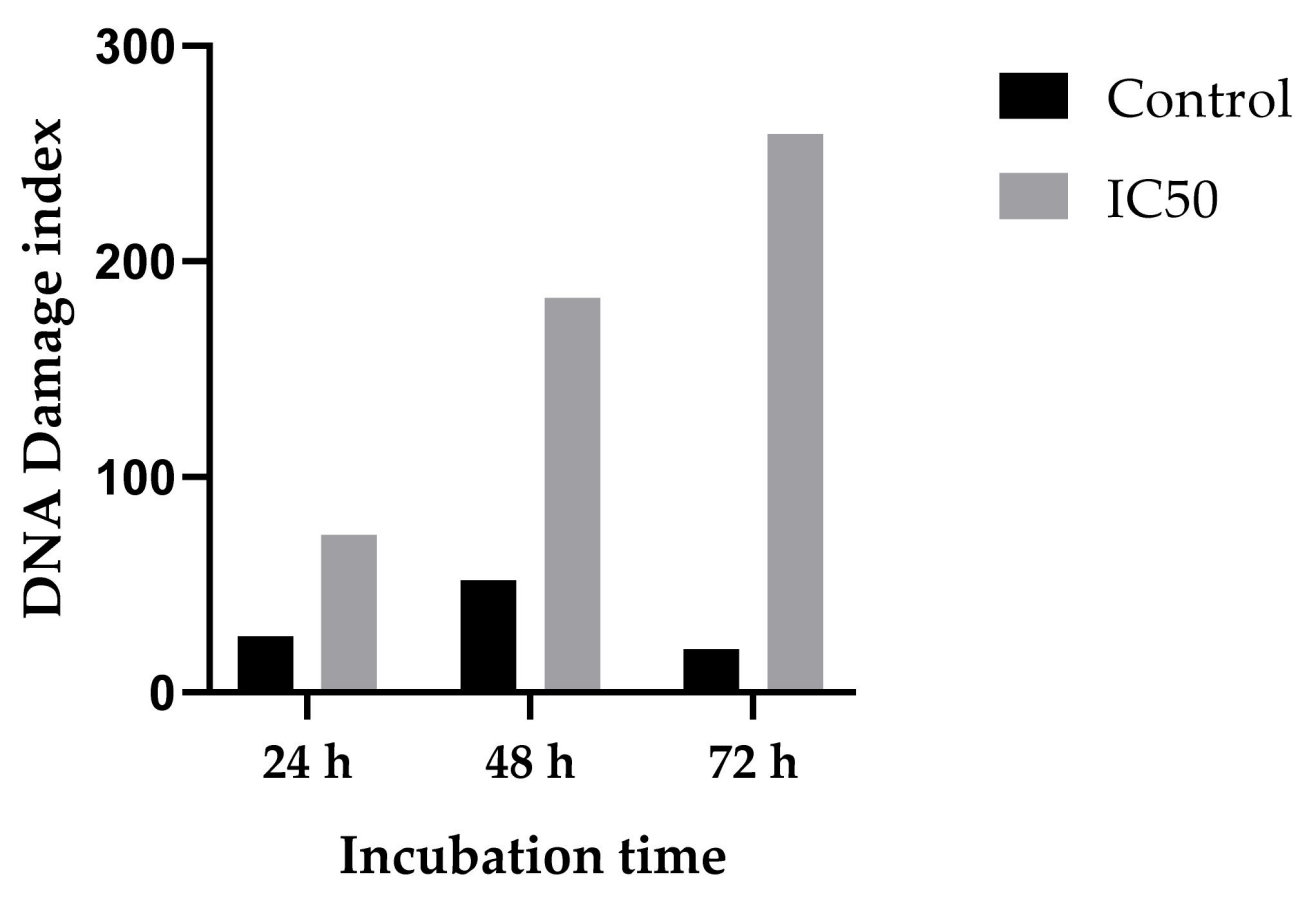
Genotoxic effect of **3** after incubation with the AGS cell line at 50 µM concentration. DNA damage index was calculated based on comet assay results. As a control, a medium with 1% DMSO was applied.

Single- and double-strand DNA breaks were measured using the comet assay. Genotoxicity of **3** was determined after 24, 48, and 72 h incubation with IC50 concentration (50 µM). As shown in Figure 12, the highest DNA damage index (DI) can be found after 72 h incubation (DI = 259). DI of the control group was within the range of 20 to 52. Following induction of DNA damage after exposure to **3**, the comet assay was used to show increased DNA damage index from 52 to 183 after 48 h, and from 20 to 259 after 72 h exposure, which proved that the damage is caused in a time-dependent manner.

### 2.4. Antimicrobial Activity Assay

According to the newest EUCAST (European Committee on Antimicrobial Susceptibility Testing)-validated publication “Clinical Breakpoints and Dosing (v.10.0)”, where *Enterococcus faecalis* ATCC 29212 is used as a quality control strain [51], the experiment was performed employing convergent parameters. The multidrug resistance of enterococci is well documented. Moreover, it is often associated with the over-expression of multidrug resistance efflux pumps, such as EmeA, which belongs to the major facilitator superfamily (MFS) and the ABC-type multidrug resistance transporters. It excludes compounds that are structurally and functionally unrelated to prevent the cellular entry of drugs from the cell or the cytoplasmic membrane. Another well-characterized efflux pump in enterococci is also a homolog of NorA and EfrAB belonging to the ATP-binding cassette (ABC) superfamily of multidrug efflux transporters [52]. Thus, the mechanisms of resistance to cell-wall active agents (i.e., beta-lactams, glycopeptides, daptomycin) that interfere with protein synthesis (aminoglycosides, oxazolidinones, streptogramins, macrolides, lincosamides, tetracyclines), and agents that interfere with nucleic acid replication, transcription, and synthesis (quinolones, rifampicin), are determined [53]. Therefore, considering the virulence and resistance of *Enterococcus spp.*, it is crucial that new compounds are analyzed against this genus, and MIC/MFC or MBC is determined.

All compounds were tested for their antimicrobial activity against a representative panel of microbials: *Pseudomonas aeruginosa* ATCC 27853, *Escherichia coli* ATCC 25922, *Staphylococcus aureus* ATCC 43300, *Streptococcus pneumoniae* ATCC 49619, *Enterococcus faecalis* ATCC 29212, *Klebsiella pneumoniae* ATCC 13883, and *Candida albicans* ATCC 10231. The in vitro antimicrobial activity was screened using the microdilution method [54] and modified Richards method [55,56,57].

For investigated compounds **2** and **3,** MIC and MBC results for *Enterococcus faecalis* 29212 were obtained; MIC/MBC/MFC was not detected in the analyzed 1024 ug/mL to 1 ug/mL of other strains. The results are presented in Table 2. For investigated compounds **2** and **3,** MIC and MBC results for *Enterococcus faecalis* 29212 were obtained; MIC/MBC/MFC was not detected in the analyzed 1024 ug/mL to 1 ug/mL of other strains. While amine **2** demonstrates weak antibacterial activity against *Enterococcus faecalis* ATCC 29212 (MBC = 1024 ug/mL; MIC = 512 ug/mL), imine **3** exhibits much stronger potential against this strain (MIC = 16 ug/mL), and, moreover, has bactericidal properties (MBC = 32 ug/mL).

## 3. Discussion

The reaction between chloromethylpyrimidine **1** and *p*-phenetidine yielded not only the expected amine **2**, but also a bright yellow byproduct separated via column chromatography, and submitted to further examination using spectroscopy techniques. The results strongly suggested that the byproduct might be a new pyrimidine Schiff base **3**, which was confirmed by devising an effective route of synthesizing **3** that exploited the coupling of aldehyde **4** with p-phenetidine in the presence of a catalytic amount of indium(III) trifluoromethanesulfonate.

**Scheme 2:**
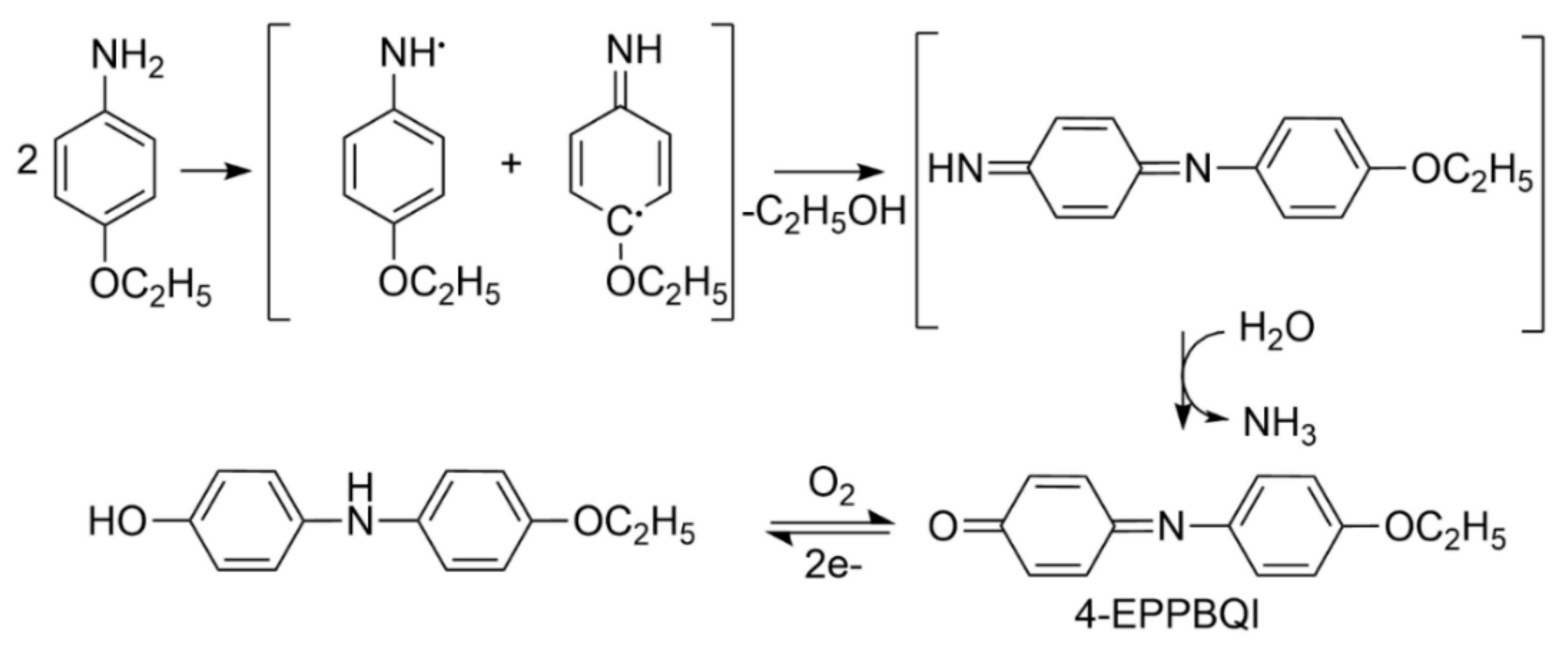
Possible mechanism of the 4-EPPBQI formation.

It appears that the formation of imine **3** as a byproduct during the reaction of chloromethylpyrimidine **1** and *p*-phenetidine can be linked with redox properties of the products of oxidation of *p*-phenetidine. There are known reactions that exploit *N*-oxides of amines, such as pyridine N-oxides [58,59,60] or another tertiary amine *N*-oxides [61,62], for the conversion of chlorides into aldehydes. On the other hand, *p*-phenetidine is suggested to form 4-(ethoxyphenyl)-*p*-benzoquinone imine (4-EPPBQI) as a major product of its oxidation (Scheme 2) [63,64]. The reactivity of benzoquinone imines has been extensively studied because of their formation during paracetamol and phenacetin metabolism as cytotoxic agents involved in several biochemical interactions [65,66]. In addition, they may undergo reactions that lead to the formation of highly reactive activated oxygen species, such as superoxide, hydrogen peroxide, or hydroxyl radicals [67], with strong oxidative properties. Thus, the proposed hypothesis of the involvement of the oxidation products of *p*-phenetidine in imine **3** formation appears entirely plausible, but requires further investigation. The employment of this reaction for the preparative obtaining of Schiff bases from chlorides is also an open issue that needs further research.

The crystal structures of **2** and **3** were determined by single-crystal X-ray diffraction. It should be noted that the imine group is attached by an N atom to a pyrimidine ring in most of the crystal structures of pyrimidine derivative Schiff bases deposited in the Cambridge Structural Database (CSD, Version 5.39) [68]. To the best of our knowledge, this is the first time that the X-ray structure of a pyrimidine derivative Schiff base, which contains a C carbon atom of the –C=N– imine bond attached to a pyrimidine ring, has been reported. Both compounds differ in conformation, which may be reflected by the dihedral angles between the pyrimidine ring and the three benzene rings. The conformation of each molecule is mainly determined by an intramolecular N–H⋯N hydrogen bond closing a six-membered ring; however, the hydrogen bond is stronger in **3** than in **2**. The major feature distinguishing the two compounds is their interaction mode and hence their packing arrangements. Intermolecular N–H⋯N hydrogen bonds connect molecules in **2** into zigzag chains, and between pyrimidine rings of molecules forming chains there are also π-π stacking interactions. These chains are further linked by weak C–H⋯O and C–H⋯F interactions into a 3D network structure. The molecules in the crystal structure of **3** are linked by a combination of C–H⋯O and C–H⋯F interactions into 2D corrugated layers.

The examination of the compounds’ impact on cells showed that both **2** and **3** do not exhibit toxic activity toward a normal cell line (L-929). Regarding cancer cell lines, amine **2** demonstrated weak anticancer activity only toward HepaRG, whereas imine **3** displayed much better anticancer properties, especially against AGS (IC50 = 50.24 µM). Annexin V/PI staining was performed to confirm the cell death pathway of **3**. Moreover, to determine the induction of DNA damage after exposure to compound **3**, the comet assay was used, which showed an increased DNA damage index. The results from annexin V/PI flow cytometry and comet assay indicate that the decrease of viability observed using neutral red uptake assay was caused by apoptotic cell death rather than growth inhibition.

For the investigated compounds, MIC and MBC results for *E. faecalis* 29212 were obtained. While amine **2** has weak antibacterial activity against *E. faecalis* ATCC 29212 (MIC = 1024 ug/mL; MBC = 512 ug/mL), imine **3** exhibits much stronger potential against this strain (MIC = 32 ug/mL) and, moreover, has bactericidal properties (MBC = 16 ug/mL).

Performed studies strongly suggested that the presence of imine instead of the amino group at position 5 of the 6-methyl-2-phenylpyrimidin-4-amine core is behind its selective and combined antineoplastic activity against AGS and bactericidal properties against *E. faecalis* of the new Shiff base **3**. Considering the fact that *E. faecalis* may be connected with the carcinogenesis of AGS, compound **3** can be viewed as a valuable structure for further optimization.

## 4. Materials and Methods

### 4.1. Chemistry

Reagents were purchased and used without purification. Lithium aluminium hydride, reagent grade 95%; pyridinium chlorochromate, 98%; thionyl chloride; silica gel, 200–400 mesh, 60 Å for column chromatography; CDCl3 for NMR spectroscopy; and *p*-phenetidine, 98%, were supplied by Sigma Aldrich, Germany. Other reagents were provided by Chempur, Poland. TLC sheets Alugram SIL G/UV254 were obtained from Mecherey-Nagel, Germany.

NMR spectra were recorded using a Bruker ARX 300 MHz NMR spectrometer. Chemical shifts (δ in ppm) are given from internal solvent-CDCl3 7.26 ppm for 1H. Abbreviations used in NMR spectra: s—singlet, d—doublet, t—triplet, q—quartet, m—multiplet. IR spectra were recorded with a Thermo Scientific USA Nicolet iS50 FT-IR using the ATR technique. Elemental analyses were performed using a Carlo-Erba NA-1500 elemental analyzer. MS spectra were recorded with a Bruker Daltonic Compact using the ESI technique.

5-[(4-*ethoxyanilino*)*methyl*]-*N*-(4-*fluorophenyl*)-6-*methyl*-2-*phenylpyrimidin*-4-*amine* (**2**)

Preparation began with 0.5 g (1.62 mmol) of [4-(4-fluoroanilino)-6-methyl-2-phenylpyrimidin-5-yl]methanol (**1**), which was placed in a round-bottom flask equipped with a reflux condenser and dissolved in 10 mL of benzene. Then 1 mL of SOCl_2_ (1.64 g, 13.78 mmol) was added, and the reaction mixture was left at room temperature for 24 h. After this time, the excess of thionyl chloride and benzene was removed under vacuum, and the solid residue was washed with 2 mL of cold benzene. Next, the crude product was inserted into a round-bottom flask equipped with a magnetic stirrer and a reflux condenser and dissolved in 10 mL of THF. Then 0.69 g (5 mmol) of *p*-phenetidine was added, and the mixture was left for 48 h at room temperature. After this time, the mixture was poured into 50 mL of cold water and then extracted three times with 10 mL of CHCl_3_. The extracts were combined and dried with over 2 g of anhydrous MgSO_4_ for 30 min. The drying agent was filtered off, and the solvent was removed under vacuum. The crude product was purified by column chromatography on silica gel using 10% ethyl acetate in CHCl_3_ as eluent and crystallized from methanol. The purity of the product was monitored by TLC using chloroform/ethyl ether (3:1) as eluent.

Product characterization: yield 0.43 g, 62%; solid beige; melting point 163–164 °C; ^1^HNMR (300 MHz, CDCl_3_): δ (ppm) 1.42 (3H, t, CH_3_), 2.54 (3H, s, CH_3_), 3.60 (1H, broad, NH), 4.01 (2H, q, CH_2_), 4.27 (2H, s, CH_2_) 6.77–8.41 (13H, m, arom.), 8.77 (1H, s, NH); FT–IR (ATR, selected lines): ν (cm^−1^) 3295 (N–H); MS (ESI) *m*/*z* [M + H]^+^ 429.2036, calculated *m*/*z* 429.2085; analysis (%), calc./found: C 72.88/72.95, H 5.88/5.56, N 13.08/12.74.

4-(4-*fluoroanilino*)-6-*methyl*-2-*phenylpyrimidine*-5-*carbaldehyde* (**4**)

Prepration began with 0.5 g (1.62 mmol) of [4-(4-fluoroanilino)-6-methyl-2-phenylpyrimidin-5-yl]methanol (**1**), which was placed in a round-bottom flask equipped with a reflux condenser and dissolved in 10 mL of dichloromethane. Next, the suspension of 0.53 g of PCC (pyridinium chlorochromate; 2.5 mmol) in 10 mL dichloromethane was added. The resulting mixture was stirred at room temperature for 3 h. The reaction mixture was then diluted with 10 mL of diethyl ether, and the solution was decanted. The remaining black resinous polymer was washed with three 5 mL portions of diethyl ether. The extracts were combined, washed with 10 mL of 2% aqueous hydrochloric acid, and two 10 mL portions of water, then dried with over 5 g of MgSO_4_, filtered, and concentrated under vacuum. The crude product was purified by column chromatography on silica gel using chloroform as eluent, and then it was crystallized from methanol. The purity of the product was monitored by TLC using chloroform as eluent.

Product characterization: yield 0.34 g, 68%; yellow solid; melting point 184 °C; ^1^HNMR (300 MHz, CDCl_3_): δ (ppm) 2.86 (3H, s, CH_3_), 7.10–8.47 (9H, m, arom.), 10.43 (1H, s, CH), 11.11 (1H, s, NH); FT–IR (ATR, selected lines): ν (cm^−1^) 3116 (N–H), 1630 (C=O); MS (ESI) *m/z* [M + H]^+^ 308.1199, calculated *m/z* 308.1193; analysis (%), calc./found: C 70.35/70.74, H 4.59/4.29, N 13.67/13.36.

5-[(4-*ethoxyphenyl*)*imino*]*methyl*-*N*-(4-*fluorophenyl*)-6-*methyl*-2-*phenylpyrimidin*-4-*amine* (**3**)

Method A

The amount of 0.3 g of 4-(4-fluoroanilino)-6-methyl-2-phenylpyrimidine-5-carbaldehyde (**3**) (0.98 mmol) was placed in a round-bottom flask equipped with a reflux condenser and dissolved in 10 mL of THF. Then 0.2 g of *p*-phenetidine (1.5 mmol) was added. The reaction mixture was refluxed for 6 h, then it was cooled down and poured into 25 mL of 2% aqueous hydrochloric acid. The solution was extracted three times with 10 mL of CHCl_3_. The extracts were combined and dried with over 2 g of anhydrous MgSO_4_ for 30 min. The drying agent was filtered off, and the solvent was removed under vacuum. The crude product was purified by column chromatography on silica gel using CHCl_3_ as eluent. The purity of the product was monitored by TLC using chloroform as eluent.

Yield: 0.28 g; 66%; yellow solid; melting point: 187–188 °C;; ^1^HNMR (300 MHz, CDCl_3_): δ (ppm) 1.46 (3H, t, CH_3_), 2.81 (3H, s, CH_3_), 4.09 (2H, q, CH_2_), 6.97–8.50 (13H, m, arom.), 8.96 (1H, s, CH), 12.75 (1H, s, NH); FT–IR (ATR, selected lines): ν (cm^−1^) 1642 (C=N); MS (ESI) *m*/*z* [M + H]^+^ 427.1882, calculated *m*/*z* 427.1929; analysis (%), calc./found: C 73.22/73.50, H 5.44/5.16, N 13.14/13.19.

Method B

The amount of 0.3 g of 4-(4-fluoroanilino)-6-methyl-2-phenylpyrimidine-5-carbaldehyde (**3**) (0.98 mmol) was placed in a round-bottom flask equipped with a reflux condenser and dissolved in 10 mL of THF. Then 0.2 g of *p*-phenetidine (1.5 mmol) and 2 mg of indium(III) trifluoromethanesulfonate were added. The reaction mixture was stirred at room temperature for 48 h and processed as in Method A. Yield: 0.40 g; 94%.

Method C

The amount of 0.3 g of 4-(4-fluoroanilino)-6-methyl-2-phenylpyrimidine-5-carbaldehyde (**3**) (0.98 mmol) was placed in a round-bottom flask equipped with a reflux condenser and suspended in 10 mL of methanol. Then 0.2 g of *p*-phenetidine (1.5 mmol) and 2 mg of indium(III) trifluoromethanesulfonate were added. The reaction mixture was stirred at room temperature for 48 h and processed as in Method A. Yield: 0.15 g; 28%.

### 4.2. X-ray Structural Studies

Compounds **2** and **3** were dissolved in 5 mL of hot methanol and acetonitrile, respectively. The prepared solution of **2** was stirred for about 15 min at 40 °C and cooled, and subsequently, 1 mL of ethanol was added. Then both solutions were closed with a perforated sealing film and placed in ambient conditions for slow evaporation. Crystals suitable for X-ray diffraction analysis were obtained after 2–3 days. The diffraction data for **2** and **3** were collected using a Kuma KM4-CCD κ-geometry automated four-circle diffractometer equipped with a CCD camera Sapphire 2 and graphite monochromatized Mo Kα radiation (λ = 0.71073 Å). The data were collected at 100(2) K using an Oxford Cryosystems cooler. The CRYSALIS program was used for data collection, cell refinement, and data reduction [69]. The data were corrected for Lorentz and polarization effects. The structures were solved using direct methods employing SHELXS-97 [70] and refined by the full-matrix least-squares on F2 with the SHELXL program [71]. All non-H atoms were refined with anisotropic displacement parameters. The N-bonded H atoms were found in the difference Fourier maps and refined with Uiso(H) = 1.2 Ueq(N). The remaining H atoms were found in the difference Fourier maps and the final refinement cycles. Next, they were treated as a riding model in geometrically optimized positions, with C–H distances in the range of 0.95–0.99 Å, and refined with Uiso(H) = 1.2 Ueq(C), except methyl groups where Uiso(H) = 1.5 Ueq(C). The figures were prepared using the DIAMOND program [72].

CCDC 2016509-2016510 contains the supplementary crystallographic data for this article. These data can be obtained free of charge from the Cambridge Crystallographic Data Centre at www.ccdc.cam.ac.uk/data_request/cif.

### 4.3. Cells and Cytotoxicity Assay

#### 4.3.1. Chemicals

Dulbecco’s modified Eagle’s medium (DMEM), Eagle’s minimum essential medium (EMEM), Ham’s nutrient mixture F12, Williams’ medium E, fetal bovine serum (FBS), Dulbecco’s phosphate-buffered saline (PBS), L-glutamine solution, trypsin-EDTA solution, penicillin-streptomycin, amphotericin B solution, MEM non-essential amino acid solution, neutral red solution (3.3 g/L), propidium iodide (PI) solution, fluorescein diacetate (FDA), Hoechst 33342, staurosporine, and Hoechst 33342 were provided by Merck (Darmstadt, Germany). Dimethyl sulfoxide (DMSO) was obtained from ALCHEM (Toruń, Poland).

#### 4.3.2. Cell Cultures and Reagent Preparation

L-929 (mouse C3H/An connective tissue), Caco-2 (human Caucasian colon adenocarcinoma), A172 (human glioblastoma), AGS (human Caucasian gastric adenocarcinoma), HepaRG (human hepatoma cell), and HeLa (human cervix epithelioid carcinoma) cell lines were obtained from the European Collection of Authenticated Cell Cultures (ECACC) and provided by Sigma Aldrich/Merck (Munich, Germany). All cell lines were cultured in complete mediums prepared according to the ECACC recommendations and incubated in a humidified atmosphere with 5% CO_2_ at 37 °C.

The 100 mM stock of **2** and **3** was prepared by dissolving the solid form in DMSO, which was dissolved to the final concentration. The concentration of DMSO in each sample did not exceed 1%.

#### 4.3.3. Neutral Red Cell Viability Screening

Neutral red uptake assay was performed according to the protocol [73]. 24 h before treatment cells were seeded in 96-well plates in 1 × 10^5^ cell/mL of complete medium. After incubation and attachment of cells, the medium was discarded, and test substances dissolved in the fresh medium were added to wells. Samples were tested after 24, 48, and 72 h incubation. A neutral red working solution (40 µg/mL) was prepared and incubated overnight. Immediately before use, it was centrifuged to remove any dye crystals. The medium from wells was aspirated, and 100 µL of neutral red was added to plates and incubated for at least 2 h in 37 °C and 5% CO_2_ in a humidified atmosphere. To inspect the absorption of neutral red to the lysosomes, microscope visualizations were performed. After incubation, cells were washed with PBS, and destain solution containing ethanol, deionized water, and glacial acetic acid (50:49:1) was added. Plates were shaken rapidly on a horizontal shaker at 37 °C. Absorption was measured using a Thermo Scientific™ (Waltham, MA, USA) Multiskan™ GO microplate spectrophotometer at 540 nm. The negative control was a medium with 1% DMSO, and the positive control was 1 µM staurosporine. Viability was calculated according to the following formula:

(1) % Viability=ODtest substance−ODblindODnegative control−ODblind×100

#### 4.3.4. Flow Cytometry

The flow cytometry analysis was performed to determine cell viability after treatment with a compound showing high proliferation inhibition in neutral red uptake assay. The test is based on FDA’s ability to pass through an intact cell membrane, where it can be hydrolyzed by cytoplasmic esterases to fluorescein, a bright green fluorophore [74]. The protocol was based on a double-staining procedure with FDA and PI [75]. For stock solutions, FDA was dissolved in DMSO and PI in H_2_O to 1 mM concentrations.

Cells were seeded in a 6-well plate with a density of 1 × 10^5^ cell/well and maintained at 37 °C under 5% CO_2_ in a humidified atmosphere. After overnight incubation, cells were treated with a compound diluted to the following concentrations: 100, 80, 40, 10, and 1 µM. Flow cytometry analyses were performed after 24, 48, and 72 h incubation. For the analysis, both floating and adherent cells were collected, centrifuged at 1600 rpm, and washed with PBS. Cells were stained with FDA with a final concentration of 1 µM in PBS, and kept in the dark for 15 min. After incubation, cells were centrifuged, resuspended in ice-cold PBS, and kept on ice until analysis. Directly before testing, samples were stained with a 1 µM PI solution for 2 min. The acquisition was performed with a CyFlow^®^ space flow cytometer, and data analyses were performed using the FloMax software (Partec, Münster, Germany). The percentage of viable cells was calculated based on FDA absorption according to the following formula:

(2) % ViabilityFDA=% live cells under experimental conditions% live cells from negative control×100

#### 4.3.5. Microscopic Observation

Morphological changes and DNA fragmentation were examined with an Olympus IX53 inverted fluorescence microscope after staining with Hoechst 33342 according to the manufacturer’s recommendations Thermo Scientific (Waltham, MA, USA). Briefly, cells were seeded in 12-well plates with a density of 1 × 10^5^ cell/mL. After overnight incubation, the test substance was added in a half-maximal inhibitory (IC50) concentration and incubated for 24, 48, and 72 h. Before microscopic observation, both adherent and floating cells were collected, centrifuged, resuspended in PBS containing 1 µg/mL Hoechst 33342 dye. After 10 min incubation in the dark, the morphology of cells and DNA were visualized using a contrast phase and a UV filter.

#### 4.3.6. Data Processing and Statistical Analysis

Neutral red uptake assay was performed in quadruple (*n* = 4). Results were calculated to mean ± standard deviation (SD) using Microsoft Office Excel 2007. The viability in negative control was compared to 100%. Statistical analyses were performed using the GraphPad Prism 8.3 for Windows (GraphPad Software, La Jolla, CA, USA, www.graphpad.com). For IC50 results, non-linear regression analyses were performed, and a sigmoidal dose-response curve (variable slope) was fitted. Differences between groups and control were tested using an ANOVA test followed by Dunnett’s post hoc analysis. The significance was set at *p* ≤ 0.05.

#### 4.3.7. Annexin V/PI Staining

A quantitative analysis of apoptosis was performed by flow cytometry with Alexa Fluor 488 annexin V and PI staining (Thermo Scientific Waltham, MA, USA). Briefly, 3 × 10^5^ cells were seeded in 6-well plates. After overnight incubation, the tested compound was added to wells in 5 × IC50 concentration (250 µM). Cells were incubated and tested after 6, 12, 18, and 24 h, according to the manufacturer’s protocol. After incubation, cells were harvested, washed with PBS, and resuspended in 1× binding buffer to a final concentration of 1–5 × 10^6^ cell/mL. Then 5 µL of Alexa Fluor 488-annexin V was added to 100 µL of cell suspension and incubated in the dark for 15 min. After incubation, the dye was removed, and cells were suspended in 1 mL of binding buffer. Directly before analysis, cells were incubated with 1 µL PI for 2 min and analyzed by flow cytometry (using CyFlow^®^ Space flow cytometer and FloMax software).

#### 4.3.8. Comet Assay

To evaluate DNA damage induced by incubation with **3**, alkaline single-cell gel electrophoresis (comet assay) was performed. Cells were seeded in 12-well plates in a density of 1 × 10^5^ cell/well. After overnight incubation, the tested compound in IC50 concentration (50 µM) was added. After incubation, cells were harvested, centrifuged, and resuspended in PBS. The cell suspension was mixed with 1% low melting point agarose and attached to the slide covered with 1% normal melting point agarose. Subsequently, slides were incubated in a buffer (pH 10.0) containing 2.5 M NaCl, 10 mM Tris-HCl buffer, 100 mM EDTA, and 1% Triton X-100 for 1 h. Then samples were transferred to alkaline electrophoresis buffer containing 0.3 M NaCl and 1mM EDTA for a 20 min incubation followed by electrophoresis performed at 25 V and 300 mA for 20 min. After electrophoresis, samples were incubated with a neutral pH buffer (0.4 M Tris-HCl, pH 7.5) for 10 min and washed with deionized water. After air-drying, samples were stained with Hoechst 33342 and visualized with a fluorescence microscope using a UV filter. The damages were visually scored from 100 randomly selected cells. Comets were classified according to tail size into five classes: class zero with no damage, class 1 with low damage, class 2 with medium damage, class 3 with high damage, and class 4, where almost all DNA was in the tail. Based on results, the damage index (DI) was calculated according to the following formula:

(3) $$DI=\left[\left(0\times n_{0}\right)+\left(1\times n_{1}\right)+\left(2\times n_{2}\right)+\left(3\times n_{3}\right)+\left(4\times n_{4}\right)\right],$$

where *n* stands for the amount of each class.

### 4.4. Antimicrobial Activity Assay

Seven reference strains from ATCC collection (*Pseudomonas aeruginosa* 27853, *Escherichia coli* 25922, *Staphylococcus aureus* 43300, *Streptococcus pneumoniae* 49619, *Enterococcus faecalis* 29212, *Klebsiella pneumoniae* 13883, and *Candida albicans* 10231) were used for the antimicrobial activity assay. For investigated strains, the following antibacterial/antifungal agents were used according to the EUCAST examination: levofloxacin, gentamicin, and amphotericin B. Obtained MIC values were the following: for *Pseudomonas aeruginosa* 27853, levofloxacin 1 µg/mL; for *Streptococcus pneumoniae* 49619, levofloxacin 0.001 µg/mL; for *Klebsiella pneumoniae* 13883, levofloxacin 0.5 µg/mL; for *Escherichia coli* 25922, gentamicin 2 µg/mL; for *Staphylococcus aureus* 43300, levofloxacin 1 µg/mL; for *Enterococcus faecalis* 29212, levofloxacin 4 µg/mL; and for *Candida albicans* 10231, amphotericin B 1 µg/mL. The antimicrobial effect of analyzed compounds was assessed according to the standard protocol using the microdilution method with spectrophotometric measurement (λ = 580 nm at the starting point and after 24 h) [76] according to ISO 20776-1:2019 [54] and modified Richards method [55,56,57]. After 24 h/37 °C in Tryptone soy broth (TSB) medium incubation, the density of bacterial suspension was measured using a densitometer, and a proper dilution was prepared (0.005MF, 5 × 105 CFU/mL), where the range 2–8 × 105 is accepted by CLSI, EUCAST [51]. Afterward, a 96-well microplate was prepared with a range from 1024 to 1 ug/mL of the specific compound solution. A positive (TSB+strain) and negative control (TSB) were also included in the test. Microplates were incubated at 37 ± 1 °C for 24 h on the shaker. After this, the aliquots of 50 uL of 1% (*m/v*) 2,3,5-triphenyltetrazolium chloride (TTC) solution were added in each well. TTC is converted into red formazan crystals in live microbial cells. MBC/MFC can be observed as the lowest concentration that did not show microbial growth by visual analysis after 24 h incubation with TTC (did not change the color to pink). The results were also confirmed by additional inoculation, where no microbial colony was detected. Thanks to both methods, MIC, MBC, or MFC can be determined.

## 5. Conclusions

One of the most serious public health problems is the expansion of antibiotic resistance of fundamental pathogenic bacteria. However, the rate of increase in the problem of the antimicrobial antibiotic resistance surveillance system is frightening. Moreover, cancer is the second serious problem where mortality has exponentially increased. Thus, the promotion of research activities concerning effective new antimicrobial and anticancer drugs is a priority at the moment.

Two newly obtained compounds, 5-[(4-ethoxyanilino)methyl]-*N*-(4-fluorophenyl)-6-methyl-2-phenylpyrimidin-4-amine (**2**) and 5-[(4-ethoxyphenyl)imino]methyl-*N*-(4-fluorophenyl)-6-methyl-2-phenylpyrimidin-4-amine (**3**), were characterized using spectroscopic and X-ray crystallography methods. The X-ray analysis showed that both compounds differ in conformation and interaction modes. In the crystal structures, the molecules are linked into chains with N–H⋯N hydrogen bonds or C–H⋯O interactions in **2** or **3**, respectively. Biological tests have shown that **3** can be recognized as an antimicrobial agent against *E. faecalis* (MIC—16 μg/mL, MBC—32 ug/mL). Moreover, IC_50_ = 53.02 µM for AGS treating by compound **3** was determined, Performed studies strongly suggested that the presence of the imine instead of the amino group at position 5 of the 6-methyl-2-phenylpyrimidin-4-amine core is behind its selective and combined antineoplastic activity against gastric adenocarcinoma, probably via the apoptotic route, and bactericidal properties against *E. faecalis* of the new Shiff base **3**. Considering the nexus between *E. faecalis* infections with the carcinogenesis of AGS, compound **3** can be viewed as a valuable structure for further optimization.

## Figures and Tables

**Table 1:** Hydrogen bonding geometrical parameters for crystals **2** and **3** (Å,°).

D—H⋯A	d(D—H)	d(H⋯A)	d(D⋯A)	<(D—H⋯A)
Compound **2**				
N4—H4⋯N5	0.860 (17)	2.277 (17)	2.9365 (18)	133.6 (14)
N5—H5⋯N1^i^	0.93 (2)	2.32 (2)	3.1358 (18)	145.4 (14)
C46—H46⋯N3	0.95	2.40	2.8894 (18)	111
C43—H43⋯O5^ii^	0.95	2.59	3.496 (2)	159
C61—H611⋯F4^iii^	0.98	2.54	3.277 (2)	132
Compound **3**				
N4—H4⋯N5	1.00 (2)	1.75 (2)	2.664 (2)	150.4 (17)
C46—H46⋯N3	0.95	2.35	2.918 (2)	118
C25—H25⋯O5^i^	0.95	2.53	3.452 (3)	165
C24—H24⋯F4^ii^	0.95	2.63	3.572 (2)	171
C53—H53⋯F4^iii^	0.95	2.64	3.298 (2)	127
Symmetry codes: **2**: (i) – x + 1/2, y, z − 1/2; (ii) – x + 1, − y + 1, z − 1/2; (iii) – x + 1/2, y + 1, z + 1/2; **3**: (i) x + 1, y, z − 1; (ii) – x + 1, y − 1/2, − z + 1/2; (iii) − x, y − 1/2, − z + 3/2.				

**Table 2:** In vitro antibacterial activity of compounds **2** and **3** expressed as a minimal inhibitory concentration (MIC) (ug/mL) and minimal bactericidal concentration (ug/mL).

Strain	*Enterococcus faecalis* ATCC 29212	*Streptococcus pneumonia* ATCC 49619	*Staphylococcus aureus* ATCC 43300	*Escherichia coli* ATCC 25922	*Klebsiella pneumonia* ATCC 13883	*Pseudomonas aeruginosa* ATCC 27853	*Candida albicans* ATCC 10231
MBC (ug/mL)							
**2**	**1024**	n/d	n/d	n/d	n/d	n/d	n/d
**3**	**32**	n/d	n/d	n/d	n/d	n/d	n/d
MIC (ug/mL)							
**2**	**512**	n/d	n/d	n/d	n/d	n/d	n/d
**3**	**16**	n/d	n/d	n/d	n/d	n/d	n/d

## Data Availability

Data is contained within the article or Supplementary Material.

## Supplementary Materials

The following are available online: Figure S1. ESI-MS spectrum of the unknown byproduct (**3**) obtained during the synthesis of compound; Figure S2. MS/MS spectrum of the unknown byproduct (**3**); Figure S3. Enlargement of the MS/MS spectrum and fragmentation pattern of structure proposed for the 11 unknown byproduct; Figure S4. Comparison of the isotopic distribution of the unknown byproduct (**3**) with the [C26H23FN4O+1]+ 13 pattern; Figure S5. IR spectrum of the unknown product (**3**); Figure S6. 1HNMR spectrum of amine; Figure S7. IR spectrum of amine **2**; Figure S8. ESI-MS spectrum of amine **2**; Figure S9. 1HNMR spectrum of aldehyde **4**; Figure S10. IR spectrum of aldehyde **4**; Figure S11. ESI-MS spectrum of aldehyde **4**; Figure S12. 1HNMR spectrum of imine **3**; Figure S13. IR spectrum of imine **3**; Figure S14. ESI-MS spectrum of imine **3**; Figure S15. Packing diagram for **2**, showing intra- and intermolecular N-H⋯N hydrogen bonds in black and 25 intermolecular interactions C-H⋯O in red and C-H⋯F in green; Figure S16. Part of the crystal structure of **3** two-dimensional structure formed *via* intermolecular interactions 27 C-H⋯O in red and C-H⋯F in green. The dashed line indicate intramolecular N-H⋯N (black) hydrogen bonds. 28 Symmetry codes: (i) x+1, y, z-1; (ii) -x+1, y-1/2, -z+1/2; (iii) -x, y-1/2, -z+3/2; Table S1. Selected crystal data and structure refinement details of compounds **2** and **3**; Table S2. Comparison of selected geometrical parameters of compounds **2** and **3**.

## Sample Availability

Samples of compound **3** are available from the authors.
