# Supplementary material for: A New Pyrimidine Schiff Base with Selective Activities against Enterococcus faecalis and Gastric Adenocarcinoma
Source: Molecules. 2021 Apr 15;26(8):2296. doi: 10.3390/molecules26082296 (PMC8071423; doi:10.3390/molecules26082296)
Supplement: Supplementary file 1 [file molecules-26-02296-s001.zip › molecules-1172301-supplementary.pdf]

molecules

Supplementary Materials

New Pyrimidine Schiff Base with Selective Activities Against *Enterococcus Faecalis* and Gastric Adenocarcionoma

Marcin Stolarczyk, Aleksandra Mikołajczyk, Aleksandra Wolska, Iwona Bryndal, Jerzy Cieplik, Tadeusz Lis and Agnieszka Matera-Witkiewicz

Table of contents:

Figure S1. ESI-MS spectrum of the unknown byproduct (**3**) obtained during the synthesis of compound **2**………2
Figure S2. MS/MS spectrum of the unknown byproduct (**3**)…………………………………………………...2
Figure S3. Enlargement of the MS/MS spectrum and fragmentation pattern of structure proposed for the unknown byproduct (**3**)………………………………………………………………………………2
Figure S4. Comparison of the isotopic distribution of the unknown byproduct (**3**) with the $[C_{26}H_{23}FN_4O+1]^+$ pattern…………………………………………………………………………………………………3
Figure S5. IR spectrum of the unknown product (**3**) …………………………………………………...3
Figure S6. ^{1}HNMR spectrum of amine **2**…………………………………………………………...4
Figure S7. IR spectrum of amine **2**……………………………………………………………………4
Figure S8. ESI-MS spectrum of amine **2**………………………………………………………………5
Figure S9. ^{1}HNMR spectrum of aldehyde **4**…………………………………………………………..5
Figure S10. IR spectrum of aldehyde **4**………………………………………………………………6
Figure S11. ESI-MS spectrum of aldehyde **4**…………………………………………………………6
Figure S12. ^{1}HNMR spectrum of imine **3**……………………………………………………………7
Figure S13. IR spectrum of imine **3**……………………………………………………………………7
Figure S14. ESI-MS spectrum of imine **3**………………………………………………………………..8
Figure S15. Packing diagram for **2**, showing intra- and intermolecular N-H···N hydrogen bonds in black and intermolecular interactions C-H···O in red and C-H···F in green………………………………………………………8
Figure S16. Part of the crystal structure of **3** two-dimensional structure formed *via* intermolecular interactions C-H···O in red and C-H···F in green. The dashed line indicate intramolecular N-H···N (black) hydrogen bonds. Symmetry codes: (i) x+1, y, z-1; (ii) -x+1, y-1/2, -z+1/2; (iii) -x, y-1/2, -z+3/2…………………………………………..9
Table S1. Selected crystal data and structure refinement details of compounds **2** and **3**……………………………...9
Table S2. Comparison of selected geometrical parameters of compounds **2** and **3**……………………………10

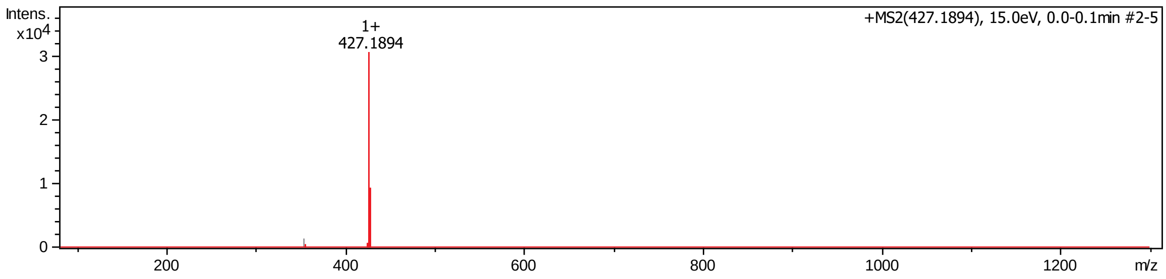

Figure S1. ESI-MS spectrum of the unknown byproduct (**3**) obtained during the synthesis of compound **2**.

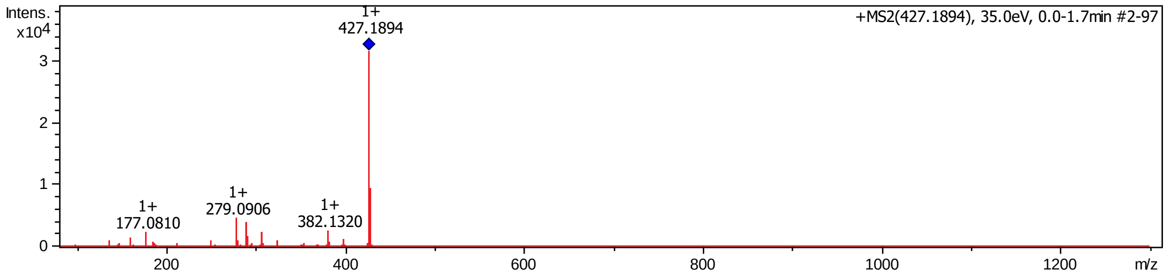

Figure S2. MS/MS spectrum of the unknown byproduct (**3**).

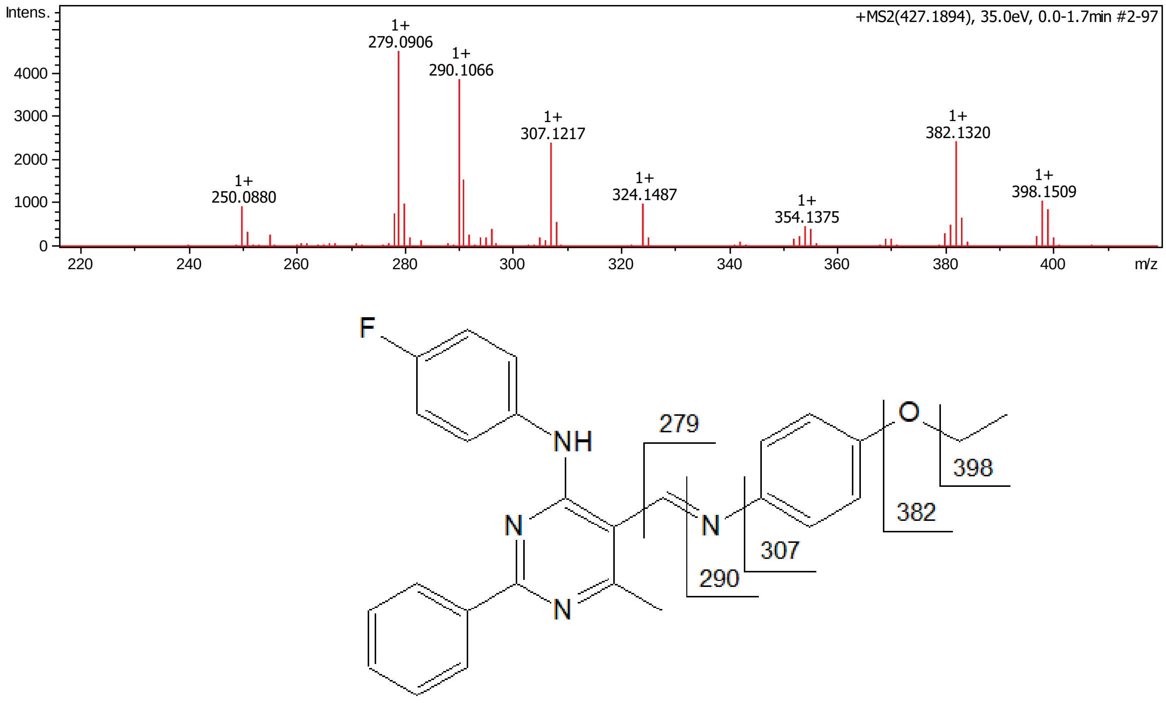

Figure S3. Enlargement of the MS/MS spectrum and fragmentation pattern of structure proposed for the unknown byproduct (**3**).

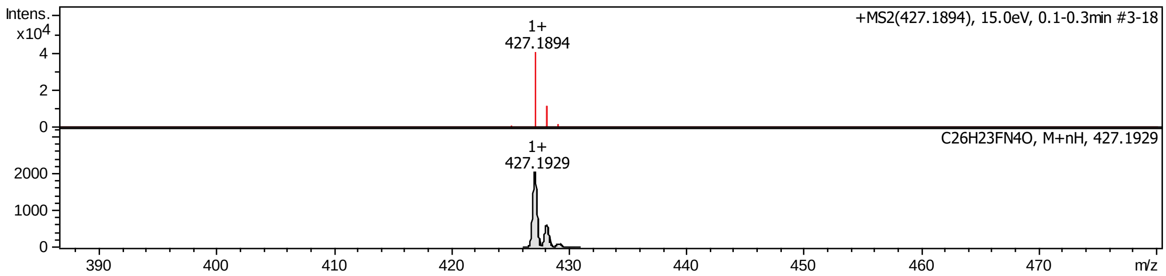

Figure S4. Comparison of the isotopic distribution of the unknown byproduct (**3**) with the $[C_{26}H_{23}FN_4O+1]^+$ pattern.

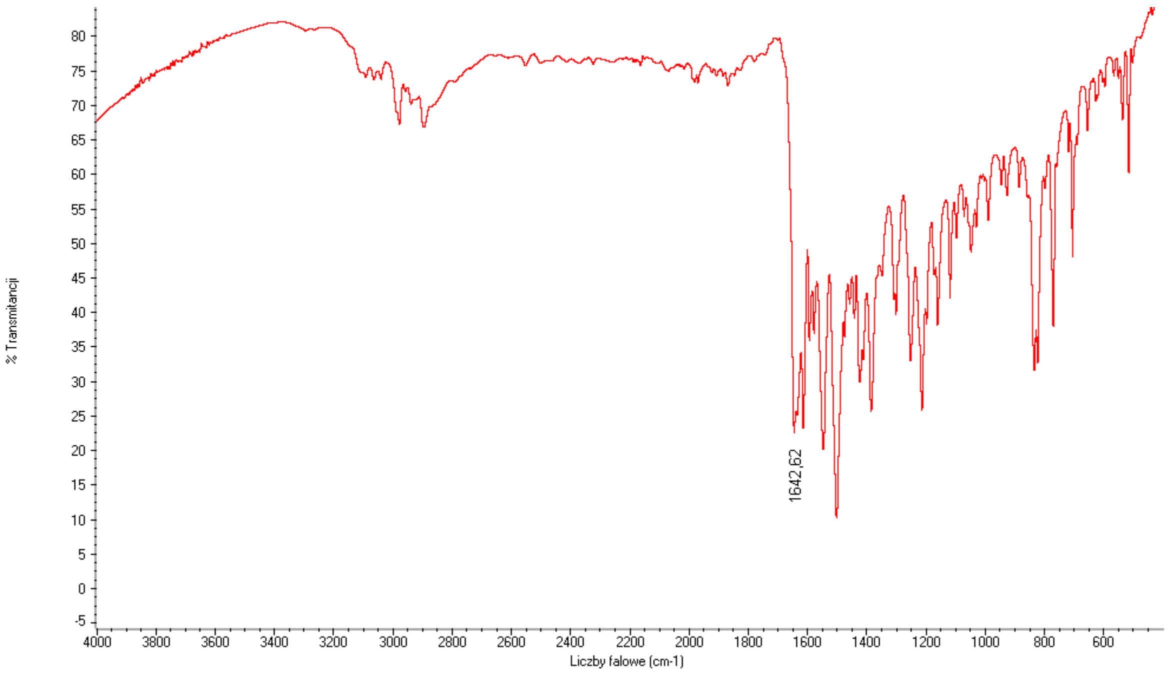

Figure S5. IR spectrum of the unknown product (**3**).

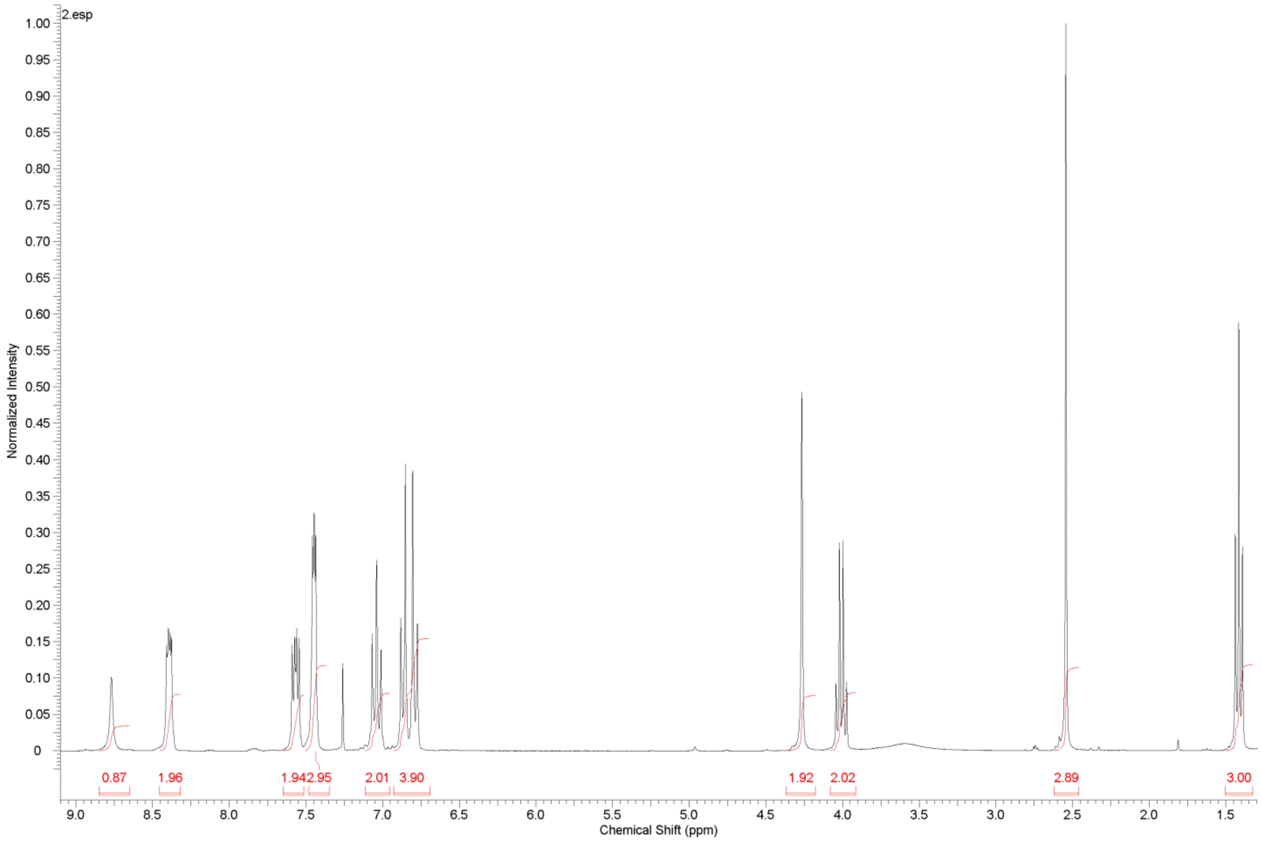

Figure S6. ^{1}HNMR spectrum of amine **2**.

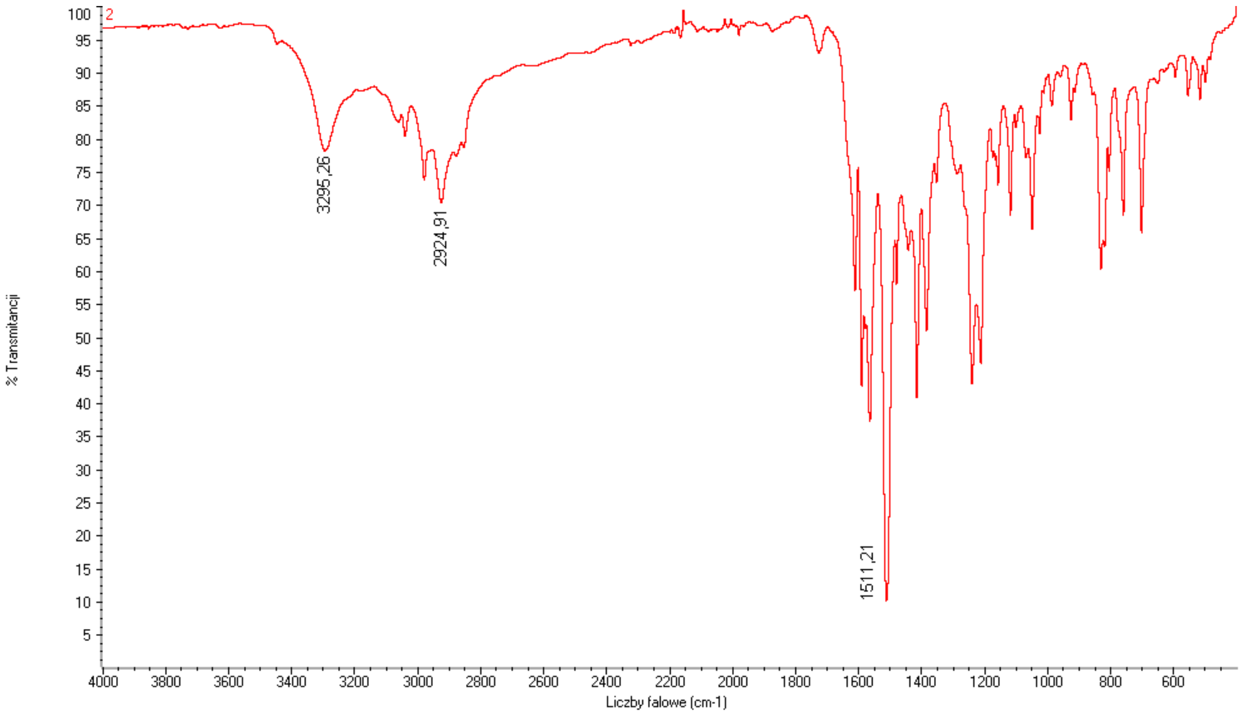

Figure S7. IR spectrum of amine **2**.

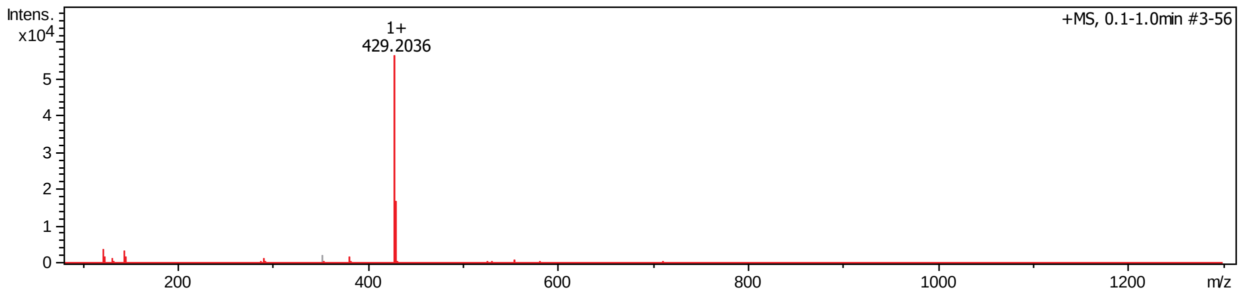

Figure S8. ESI-MS spectrum of amine **2**.

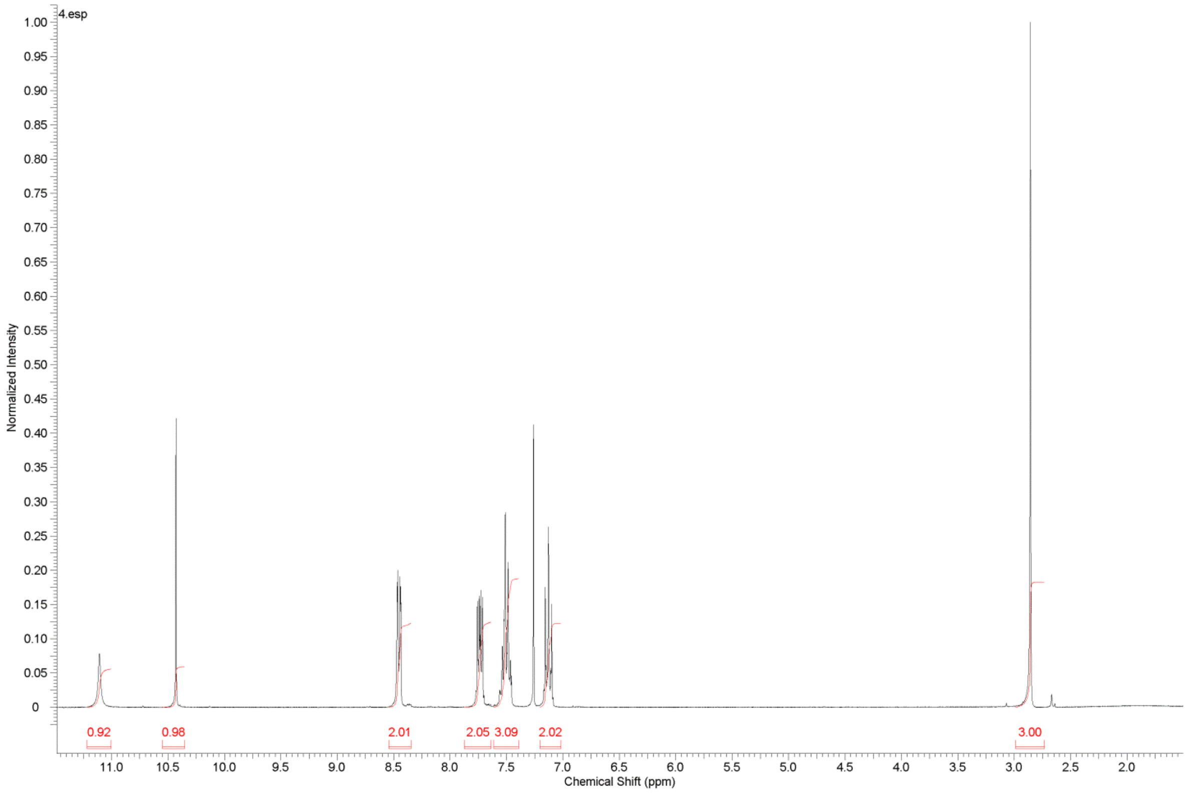

Figure S9. ^{1}HNMR spectrum of aldehyde **4**.

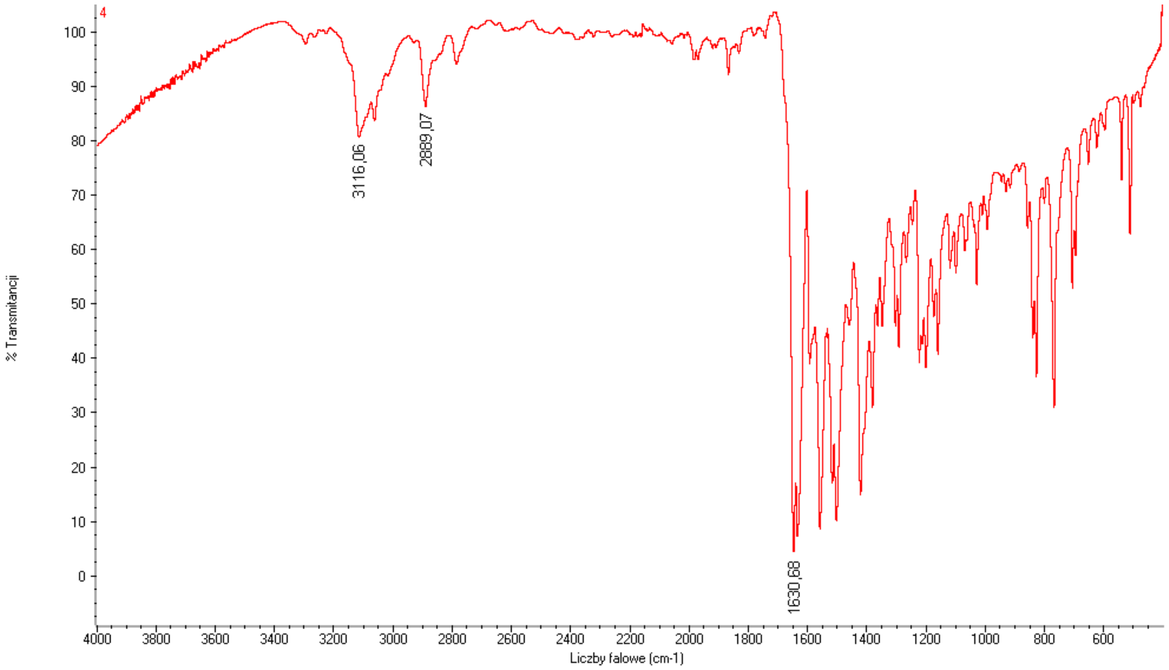

Figure S10. IR spectrum of aldehyde **4**.

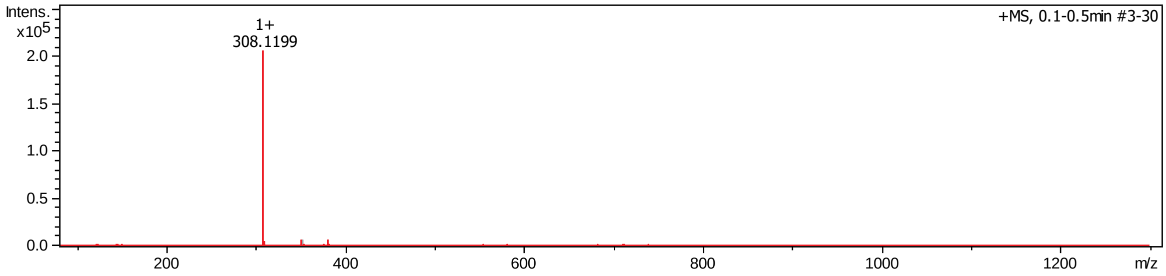

Figure S11. ESI-MS spectrum of aldehyde **4**.

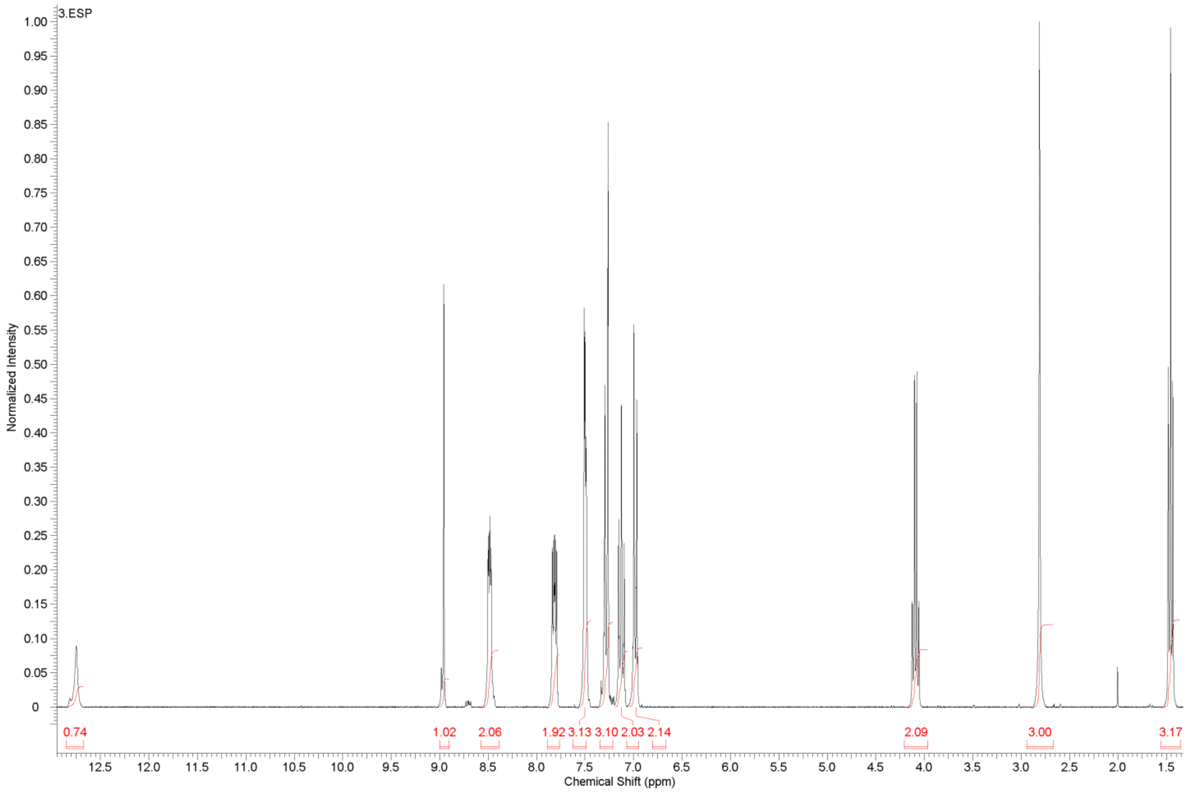

Figure S12. ^{1}HNMR spectrum of imine **3**.

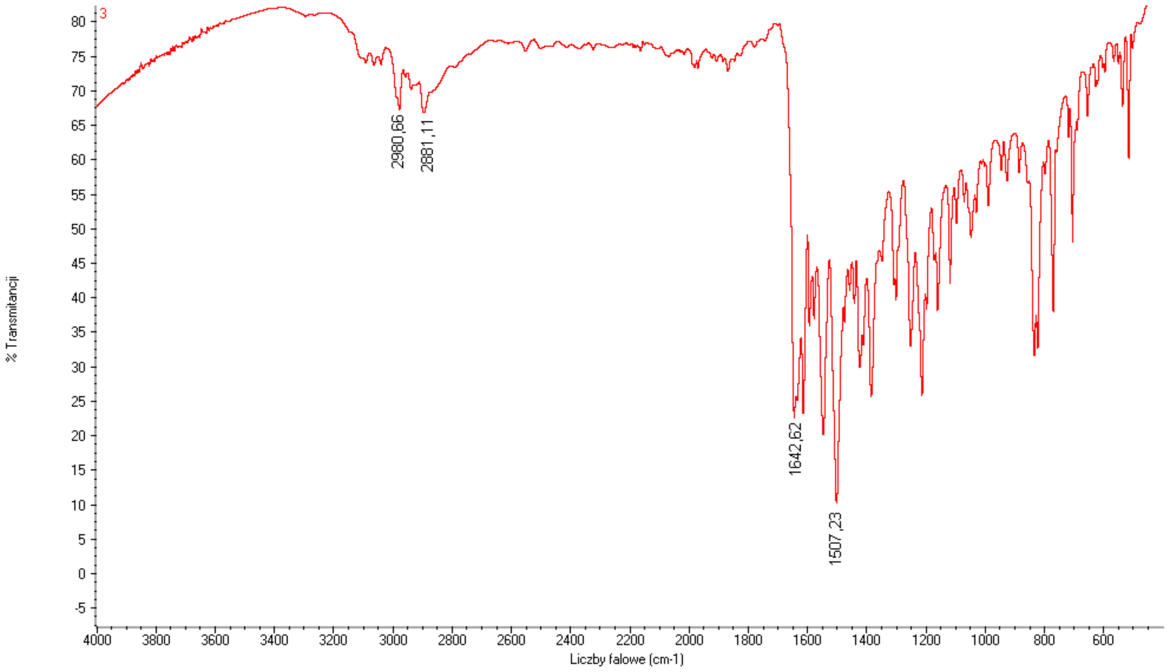

Figure S13. IR spectrum of imine **3**.

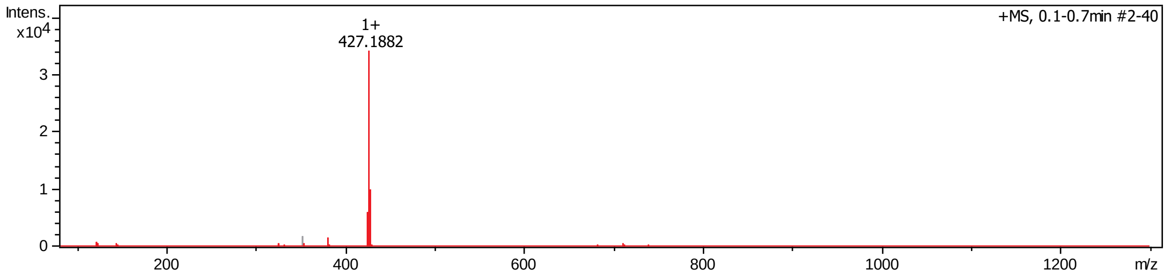

Figure S14. ESI-MS spectrum of imine **3**.

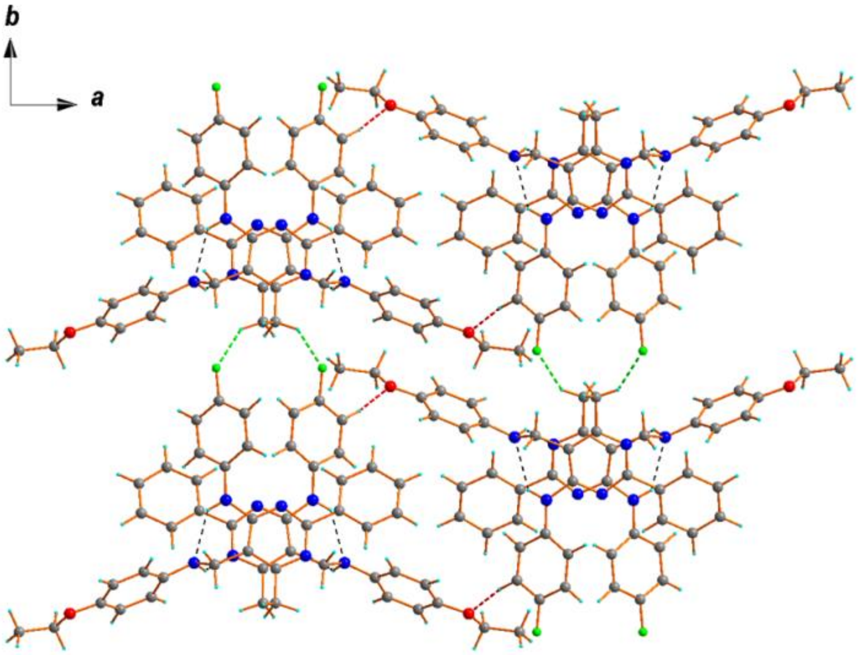

Figure S15. Packing diagram for **2**, showing intra- and intermolecular N-H···N hydrogen bonds in black and intermolecular interactions C-H···O in red and C-H···F in green.

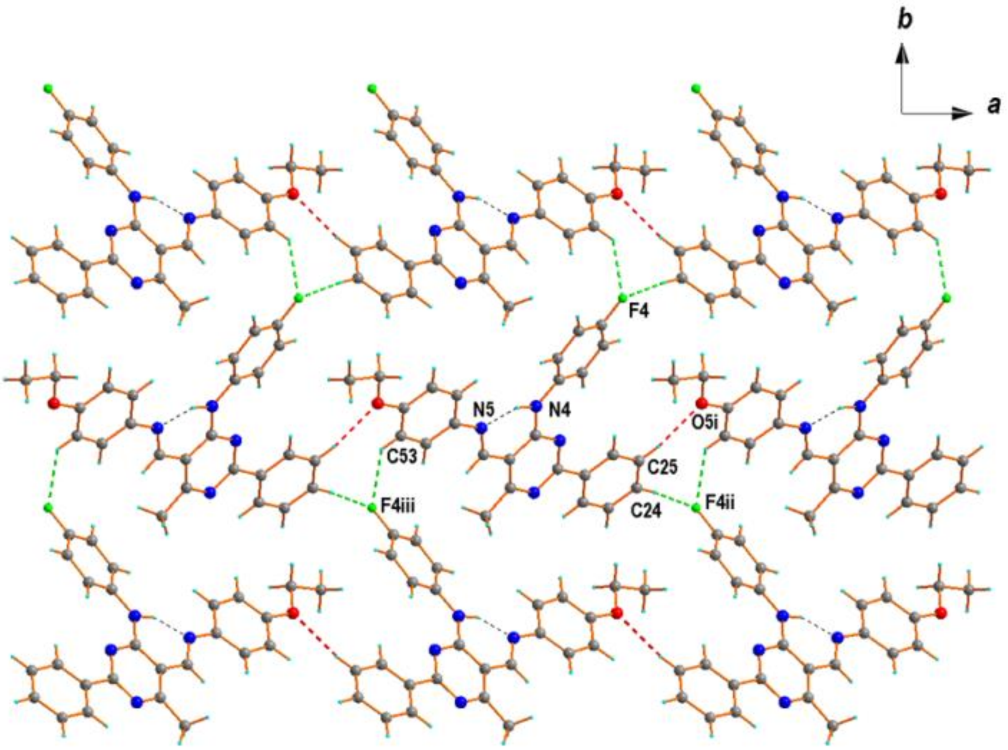

Figure S16. Part of the crystal structure of **3** two-dimensional structure formed *via* intermolecular interactions C-H···O in red and C-H···F in green. The dashed line indicate intramolecular N-H···N (black) hydrogen bonds. Symmetry codes: (i) x+1, y, z-1; (ii) -x+1, y-1/2, -z+1/2; (iii) -x, y-1/2, -z+3/2.

Table S1. Selected crystal data and structure refinement details of compounds **2** and **3**.

	**2**	**3**
Empirical formula	$C_{26}H_{25}FN_4O$	$C_{26}H_{23}FN_4O$
Formula weight	428.50	426.48
Temperature/K	100(2)	100(2)
Crystal system	orthorhombic	monoclinic
Space group	$Pca2_1$	$P2_1/c$
a/Å	26.599 (6)	12.933 (4)
b/Å	11.680 (3)	16.735 (5)
c/Å	7.277 (2)	9.547 (3)
β/°	90	90.93 (3)
V/Å^3	2260.9 (10)	2066.0 (11)
Z	4	4
F(000)	904	896
θ range (°)	2.9-30.1	2.9-30.1
Total reflections	31641	25918
Observed reflections	5294	3185
S	1.01	1.00
R_1 *(obs. data)*	0.044	0.061
wR_2 *(all data)*	0.106	0.145
CCDC	2016509	2016510

Table S2 . Comparison of selected geometrical parameters of compounds **2** and **3**.

	2	3		2	3
F4—C44	1.3659 (17)	1.367 (2)	C2—C21	1.4895 (19)	1.488 (2)
C41—N4	1.4117 (17)	1.413 (2)	N1—C6	1.3548 (18)	1.350 (2)
N4—C4	1.3626 (18)	1.358 (2)	C2—N1	1.3373 (18)	1.345 (2)
C4—N3	1.3350 (17)	1.348 (2)	C57—N5	1.4612 (19)	1.288 (2)
N3—C2	1.3353 (17)	1.337 (2)	N5—C51	1.4132 (18)	1.420 (2)
C5—C6	1.381 (2)	1.392 (2)	C54—O5	1.3737 (17)	1.374 (2)
C5—C57	1.512 (2)	1.457 (2)	O5—C58	1.431 (2)	1.421 (2)
C4—N4—C41	127.61 (12)	130.15 (15)	N1—C6—C5	122.58 (12)	122.63 (16)
N3—C4—N4	118.61 (12)	119.24 (16)	N5—C57—C5	111.79 (12)	122.59 (17)
N3—C4—C5	121.92 (12)	121.76 (16)	C51—N5—C57	116.02 (12)	122.06 (16)
N4—C4—C5	119.47 (13)	118.99 (15)	C52—C51—N5	121.03 (13)	125.58 (16)
C4—N3—C2	117.16 (11)	116.77 (15)	C56—C51—N5	120.96 (13)	116.63 (16)
C4—C5—C57	120.83 (13)	123.58 (16)	O5—C54—C53	124.54 (14)	114.85 (16)
N3—C2—N1	125.99 (12)	126.40 (16)	O5—C54—C55	115.91 (13)	125.43 (17)
C2—N1—C6	116.43 (12)	116.65 (16)	C54—O5—C58	117.67 (13)	118.19 (15)
C42—C41—N4—C4	155.49 (14)	-163.19 (18)	C2—N1—C6—C5	−0.13 (18)	2.8 (3)
C46—C41—N4—C4	−29.0 (2)	17.7 (3)	C2—N1—C6—C61	−178.99 (11)	-175.92 (16)
C41—N4—C4—N3	−13.3 (2)	-1.3 (3)	C4—C5—C6—N1	−0.45 (19)	-0.1 (3)
C41—N4—C4—C5	167.08 (13)	177.38 (16)	C57—C5—C6—N1	−175.91 (13)	178.54 (17)
N4—C4—N3—C2	179.48 (12)	-178.79 (16)	C57—C5—C6—C61	2.9 (2)	-2.8 (3)
C5—C4—N3—C2	−0.94 (18)	2.8 (3)	C6—C5—C57—N5	−124.54 (14)	-178.06 (17)
N3—C4—C5—C6	1.01 (18)	-2.6 (3)	C4—C5—C57—N5	60.22 (17)	1.0 (3)
N4—C4—C5—C6	−179.42 (12)	179.10 (16)	C5—C57—N5—C51	174.27 (12)	176.65 (16)
N3—C4—C5—C57	176.58 (12)	178.65 (17)	C57—N5—C51—C52	−135.55 (15)	173.46 (17)
N4—C4—C5—C57	−3.84 (19)	0.0 (3)	C57—N5—C51—C56	47.23 (19)	-9.0 (3)
C4—N3—C2—N1	0.31 (19)	-0.6 (3)	C53—C54—O5—C58	−12.1 (2)	-1.8 (3)
C4—N3—C2—C21	179.08 (11)	-178.63 (15)	C55—C54—O5—C58	168.45 (14)	177.46 (16)
N3—C2—N1—C6	0.22 (19)	-2.3 (2)	C54—O5—C58—C59	−170.23 (14)	-173.83 (16)
C21—C2—N1—C6	−178.53 (11)	175.93 (15)			
